# Bifunctional activation of amine-boranes by the W/Pd bimetallic analogs of “frustrated Lewis pairs”†

**DOI:** 10.1039/d0sc06114j

**Published:** 2021-01-15

**Authors:** Elena S. Osipova, Ekaterina S. Gulyaeva, Evgenii I. Gutsul, Vladislava A. Kirkina, Alexander A. Pavlov, Yulia V. Nelyubina, Andrea Rossin, Maurizio Peruzzini, Lina M. Epstein, Natalia V. Belkova, Oleg A. Filippov, Elena S. Shubina

**Affiliations:** A.N. Nesmeyanov Institute of Organoelement Compounds, Russian Academy of Sciences (INEOS RAS) Vavilova Str. 28 119991 Moscow Russia shu@ineos.ac.ru nataliabelk@ineos.ac.ru; Istituto di Chimica dei Composti Organometallici – Consiglio Nazionale delle Ricerche (ICCOM – CNR) Via Madonna del Piano 10 50019 Sesto Fiorentino Italy maurizio.peruzzini@iccom.cnr.it

## Abstract

The reaction between basic [(PCP)Pd(H)] (PCP = 2,6-(CH_2_P(*t*-C_4_H_9_)_2_)_2_C_6_H_4_) and acidic [LWH(CO)_3_] (L = Cp (**1a**), Tp (**1b**); Cp = η^5^-cyclopentadienyl, Tp = κ^3^-hydridotris(pyrazolyl)borate) leads to the formation of bimolecular complexes [LW(CO)_2_(μ-CO)⋯Pd(PCP)] (**4a**, **4b**), which catalyze amine-borane (Me_2_NHBH_3_, ^*t*^BuNH_2_BH_3_) dehydrogenation. The combination of variable-temperature (^1^H, ^31^P{^1^H}, ^11^B NMR and IR) spectroscopies and computational (ωB97XD/def2-TZVP) studies reveal the formation of an η^1^-borane complex [(PCP)Pd(Me_2_NHBH_3_)]^+^[LW(CO_3_)]^−^ (**5**) in the first step, where a BH bond strongly binds palladium and an amine group is hydrogen-bonded to tungsten. The subsequent intracomplex proton transfer is the rate-determining step, followed by an almost barrierless hydride transfer. Bimetallic species **4** are easily regenerated through hydrogen evolution in the reaction between two hydrides.

## Abstract 2

Bimetallic complexes [LW(CO)_2_(μ-CO)⋯Pd(PCP)] cooperatively activate amine-boranes for their dehydrogenation *via* N–H proton tunneling at RDS and H_2_ evolution from two neutral hydrides.

## Graphical abstract

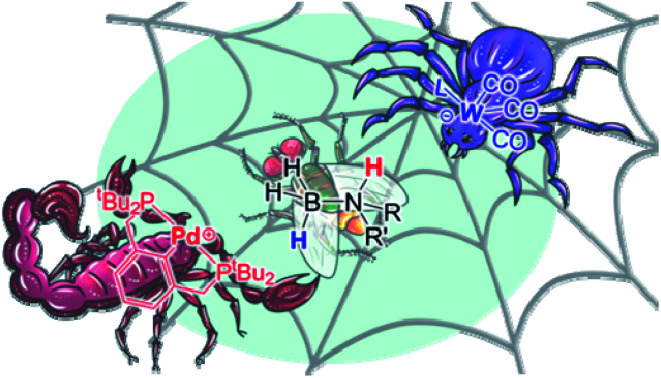

## Introduction

Catalytic processes occurring under the action of “frustrated Lewis pairs” (FLPs) have been intensively sought after during the past decade.^1,2^ Heterolytic cleavage of H_2_ by main group intermolecular FLPs has been proposed to occur through an ‘encounter complex’ where the Lewis acid and Lewis base are in close proximity, but non-bonding, and H_2_ is accommodated between the two centers prior to heterolysis. DFT metadynamics studies for the prototypical FLP system P(Mes)_3_/B(C_6_F5)_3_ gave evidence for the H_2_ polarization followed by the rate-determining hydride transfer to B and proton transfer to P.^3^

The heterolytic cleavage of H_2_ into a proton and a hydride is a crucial step in many chemical and biochemical processes such as, *e.g.*, hydrogen oxidation by hydrogenases, transition metal-catalyzed hydrogenation of C=O bonds, or metal-catalyzed hydrogen oxidation in energy conversion reactions. From this point of view, the nomenclature of FLPs has many parallels in metal–ligand cooperation (bifunctional) catalysis, where the two sites of cooperation are typically metal- and ligand-based.^1,4,5^ Closely related to this concept is also an area of bimetallic catalysis, wherein two metal sites demonstrate cooperativity in fundamental catalytic reactions.^6^

**Scheme 1:**
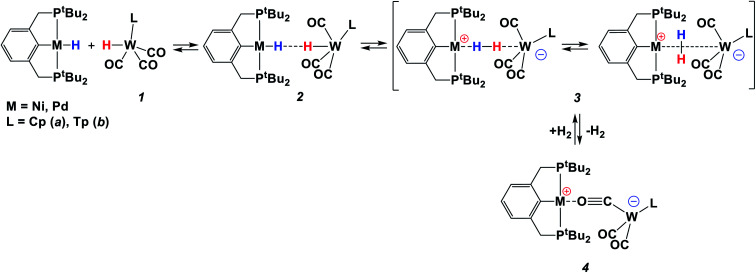
Reaction between two metal hydrides, (PCP)PdH and **1**.

The species featuring an H_2_ molecule poised between the Lewis acid and Lewis base centers have been found by us in previous work as the intermediate of dihydrogen evolution in the reaction of two neutral transition metal hydrides of different polarities. Dihydrogen bonding (DHB, M–H^*δ*−^⋯^*δ*+^H–M′; M–H = (PCP)NiH, (PCP)PdH; M′–H = CpWH(CO)_3_ (**1a**); PCP = 2,6-C_6_H_3_(CH_2_P^*t*^Bu_2_)_2_) precedes the concerted proton and hydride transfer,^7^ eventually yielding the bimetallic isocarbonyl bridging M/W bimetallic complex **4** (Scheme 1).^8,9^ Up to date, there are only a few examples of the attempted use of such systems in catalysis. CpMoH(CO)_3_ has been used as a proton source in Rh-catalyzed hydroformylation.^10^ More recently, the switch of selectivity between migratory insertion *versus* C–H activation has been shown for the reaction of [(^*i*Pr^POCOP)Ni]^+^ (^*i*Pr^POCOP = 2,6-C_6_H_3_(OP^*i*^Pr_2_)_2_) with phenylacetylene in the presence of [CpW(CO)_3_]^−^ presumably acting as a proton shuttle.^11^ However, this reaction was performed only in a stoichiometric regime.

In the field of chemical hydrogen storage, lightweight B/N-containing inorganic hydrides (ammonia borane, amine-boranes, and hydrazine boranes) have gained increasing attention in the last few years as sources of carbon-free and high-purity H_2_ for renewable energy applications.^12–14^

Metal-catalyzed dehydrogenation became a well-developed route for H_2_ production from amine-boranes^15^ and new studies keep appearing in the literature aiming to optimise the reaction conditions and to control the selectivity in product distribution as dehydrogenative coupling not only produces H_2_ as an energy source but also B–N-containing materials.^16,17^ The metal-free catalysts including FLPs are also known but far less numerous.^18^ Although direct comparison between different catalysts is somewhat ambiguous due to the difference in the reaction conditions, RuH(PMe_3_){N(CH_2_CH_2_P^i^Pr_2_)_2_} can be considered as the best catalyst for BH_3_NH_3_ dehydrogenation (TOF_max_ 72 000 h^−1^)^19^ and (POCOP)IrH_2_ (TOF 2400 h^−1^)^12^ and Rh(Xantphos-^i^Pr)H (1500 h^−1^)^20^ for BH_3_NMeH_2_ dehydrogenation, whereas a cationic zirconocene–phosphinoaryloxide complex [Cp_2_ZrOC_6_H_4_P^*t*^Bu_2_]^+^ which can be described as an early transition-metal-containing FLP gives TOF 600 h^−1^ for BH_3_NMe_2_H dehydrogenation.^21^

The simultaneous presence of protic and hydridic H atoms within the same amine-borane molecule makes them alternative reagents in transfer hydrogenation which attracts increased research interest.^22^ It also triggers an extended dihydrogen bonding (DHB) throughout their solid-state structure.^23^ DHB is preliminary to H_2_ evolution occurring after a simple thermal treatment or in a catalytic fashion.^15,24^ Given the analogy between protic and hydridic H atom co-existence and heterolytic H_2_ cleavage, the latter is intimately related to B/N inorganic hydride activation. Herein, we show for the first time that the bimetallic systems reported above act as homogeneous amine-borane dehydrogenation catalysts and explore the impact of hydride and proton transfer steps in the reaction mechanism using **1a** in comparison with its more acidic analog TpWH(CO)_3_ (**1b**; Tp = κ^3^-hydridotris(pyrazolyl)borate) as proton donating hydrides (Scheme 1).

## Results and discussion

### Pairwise interaction of two metal hydrides

**Fig. 1:**
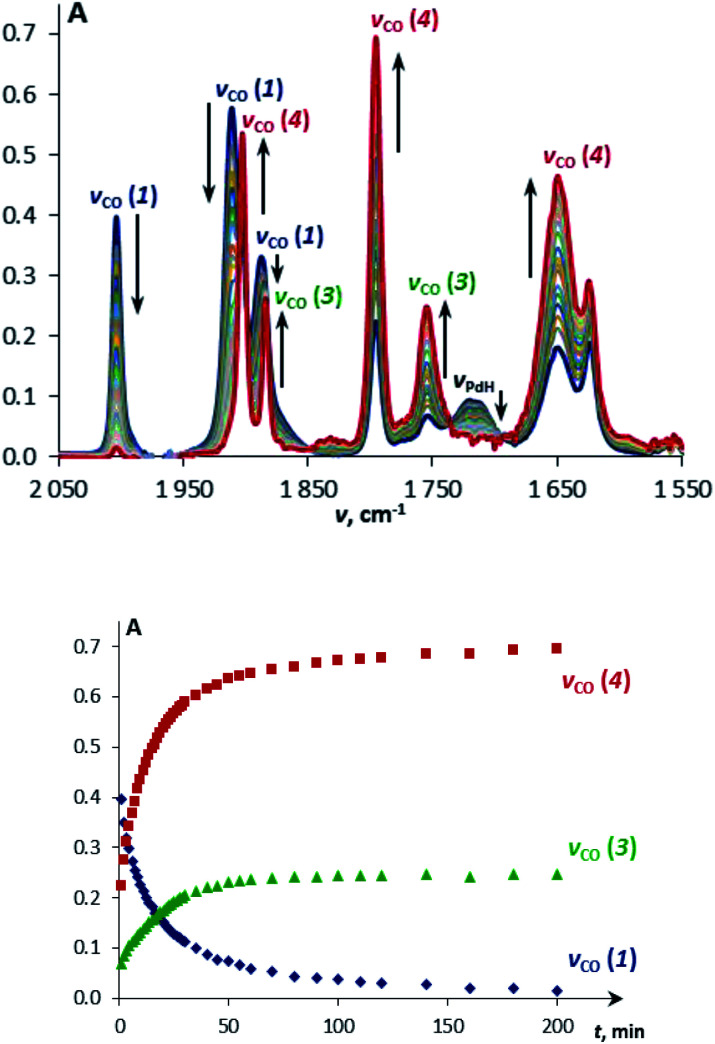
IR spectra (top) and the corresponding kinetic curves (bottom) of a mixture of TpWH(CO)_3_ (**1a**, *c* = 0.003 M) and (PCP)PdH (*c* = 0.045 M) at 240 K in THF.

**Fig. 2:**
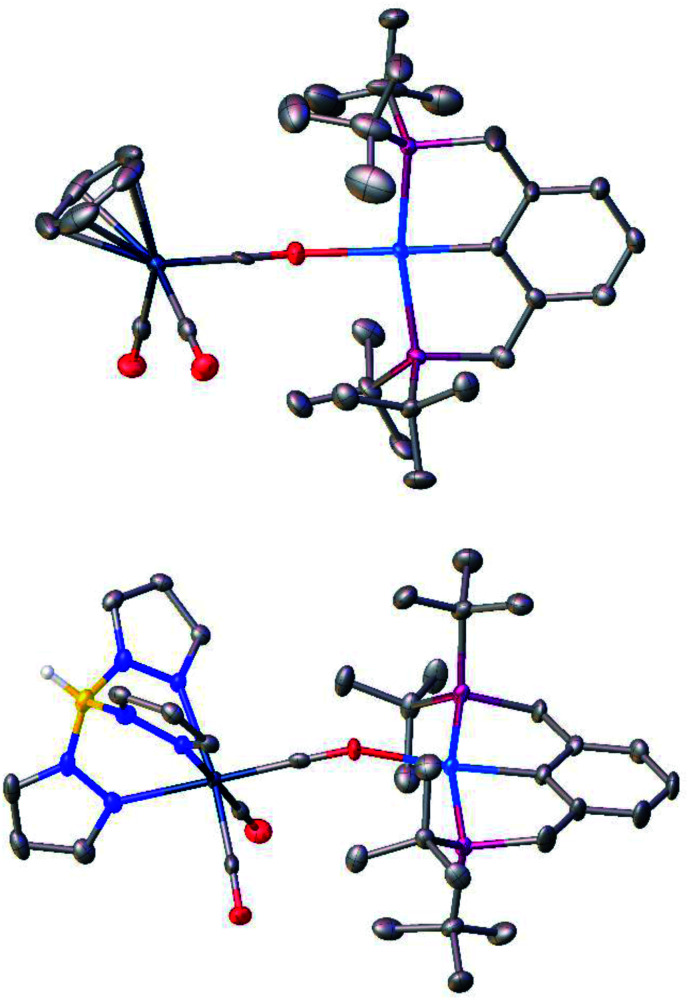
Molecular structures of bimetallic products **4a** (top) and **4b** (bottom). Atom color code: white, H; gray, C; yellow, B; red, O; blue, N; navy, W; pink, P; and cyan, Pd. Thermal ellipsoids are given at 50% probability level; hydrogen atoms except for the BH group in **4b** are omitted for clarity.

The reaction of TpWH(CO)_3_ (**1b**) with (PCP)PdH follows the same mechanism (Scheme 1) as that of CpWH(CO)_3_ (**1a**). The IR spectra obtained for the mixture of **1b** with a 1.5-fold excess of (PCP)PdH in pure THF at 200 K show two new *ν*_CO_ bands of the intermediate **3b** at 1884 and 1754 cm^−1^, which disappear gradually upon temperature increase (Scheme 1 and Fig. S1†). At the same time, the bands at 1650, 1796, and 1901 cm^−1^ of the H_2_ evolution product – isocarbonyl complex **4b** – increase in intensity. In a closed system, the reaction goes to equilibrium (Fig. 1). When the reaction mixture was allowed to reach equilibrium at, *e.g.*, 240 K and then warmed to 290 K, the intensity decrease of the *ν*_CO_ bands of the ionic intermediate **3b** and a slight increase of the *ν*_CO_ bands of the initial hydride **1b** are observed. This indicates the shift of the proton transfer equilibrium **1** ↔ **3** to the left. At the same time, the *ν*_CO_ intensity of complex **4b** increases due to the hydrogen evolution and right shift of the **3** ↔ **4** equilibrium (Fig. S2†).^25^ The IR monitoring of the reaction kinetics showed that the proton transfer is the rate-determining step of the overall process and the rate constant values are larger for TpWH(CO)_3_ (**1b**) than those with CpWH(CO)_3_ (**1a**): at 240 K *k*_obs_ = 0.202 mol^−1^ s^−1^ for **1b** and 0.140 mol^−1^ s^−1^ for **1a**. This gives Gibbs free energy values of Δ*G*^‡^_298 K_ = 16.5 ± 0.1 kcal mol^−1^ for **1b** and 16.9 ± 0.1 kcal mol^−1^ for **1a**. Complexes **4** reversibly capture molecular hydrogen similarly to FLPs. In a closed system, the reverse cooling from 290 K to 240–200 K restores the original spectroscopic picture. The crystal structures of both bimetallic complexes **4** (Fig. 2) appeared to be very similar to those reported for the related Ni-containing complexes.^8,9,11^ The larger steric volume of the Tp ligand leads to a different orientation of the [TpW(CO)_3_] fragment: rotation around the Pd–O bond places terminal CO ligands on one side of the [(PCP)Pd] plane.

### Interaction with dimethylamine-borane (DMAB)

Since the bifunctional ionic pairs [LW(CO)_2_(μ-CO)⋯M(PCP)] (**4**) reversibly bind hydrogen, we hypothesised that they could interact with amine-boranes which are typical bifunctional molecules (RR′NHBH_3_; R = H, Me; R′ = H, Me, ^*t*^Bu). We used substituted amine-boranes for these studies because they produce boron-containing reaction products and intermediates soluble in organic solvents.^26^ Besides, Me_2_NHBH_3_ usually reacts slower than ammonia borane (NH_3_BH_3_).^27^

**Scheme 2:**
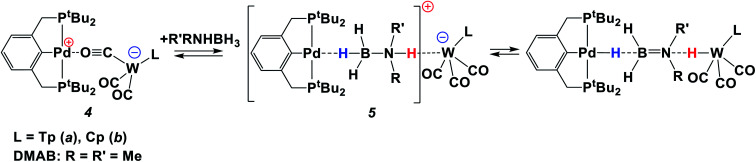
Reaction between the ionic pair **4** and amine-borane.

The addition of excess Me_2_NHBH_3_ (DMAB, 3.3 equiv.) to the bimetallic complexes **4** in THF at 270 K disrupts the inter-ion interaction due to the simultaneous coordination of DMAB molecules between the two metals. The decrease of *ν*_CO_ bands of **4** and the appearance of two new *ν*_CO_ bands of a new ionic complex **5** (Table 1) are observed in the IR spectra (Fig. S3†). These changes are reversible; the equilibrium shifts toward **5** upon cooling. Changing the solvent to less polar toluene allows the reaction to move one step forward: the bands of LWH(CO)_3_ (**1**) and (PCP)PdH (*ν*_PdH_ 1717 cm^−1^) appear in the IR spectrum (Fig. S4†), confirming the occurrence of proton and hydride transfer and the formation of neutral molecules. The hydride transfer from the boron atom to palladium is confirmed by the synchronous decrease of the DMAB band intensity (*ν*_BH_ 2364 cm^−1^) and the *ν*_PdH_ growth on going from 260 K to 190 K (Fig. S5†). Warming the mixture from 190 to 260 K restores the original spectral picture, further confirming the existence of the equilibrium shown in Scheme 2. Definitely, the neutral trimolecular complex should be unfavorable due to the high entropy effect (see DFT calculations below), but it is conserved at low temperatures when *T*Δ*S* contribution to the Gibbs energy is diminished.

**Table 1:** *ν*_CO_ vibrations (cm^−1^) of the metal complexes in THF and toluene at 298 K

Complex		*ν*_CO_, cm^−1^ in THF	*ν*_CO_, cm^−1^ in toluene
**1a**	CpWH(CO)_3_	2018, 1924	2020, 1926
**1b**	TpWH(CO)_3_	2003, 1910, 1888	2006, 1914, 1891
**3a**, **5a**	[CpW(CO)_3_]^−^	1891, 1775	1887, 1782
**3b**, **5b**	[TpW(CO)_3_]^−^	1884, 1754	1862, 1767
**4a**	[CpW(CO)_2_(μ-CO)⋯Pd(PCP)]	1910, 1819, 1660	1907, 1814, 1656
**4b**	[TpW(CO)_2_(μ-CO)⋯Pd(PCP)]	1901, 1796, 1650	1904, 1798, 1644

The behavior of two ionic complexes **4a** and **4b** is qualitatively the same. However, the proton transfer from DMAB to **4a** is easier; the tungsten hydride **1a** is formed in a larger quantity, in agreement with the higher basicity of the [CpW(CO)_3_]^−^ anion: p*K*_a_ of the conjugated acid LWH(CO)_3_ in CH_3_CN is 16.1 (ref. 28) and 14.4 (ref. 29) for **1a** and **1b**, respectively.

Thus, at low temperature (below 270 K), the DMAB molecule becomes “arrested” between the two metal ions of **4**. The bifunctional nature of **4** allows the proton transfer from the N–H moiety to the Lewis base center on tungsten and the hydride transfer from the B–H moiety to the Lewis acid center on palladium. The position of the equilibrium (Scheme 2) depends on the solvent, temperature, and basicity of the [LW(CO)_3_]^−^ anion. Interestingly, neither (PCP)PdH, LWH(CO)_3_, [LW(CO)_3_]^−^ (obtained by the reaction of LWH(CO)_3_ with NaHMDS) or [(PCP)Pd]^+^ (ref. 27) taken individually shows any activity in DMAB dehydrogenation. This confirms the crucial role played by the bimetallic complex in the process.

### Catalytic DMAB dehydrogenation

**Fig. 3:**
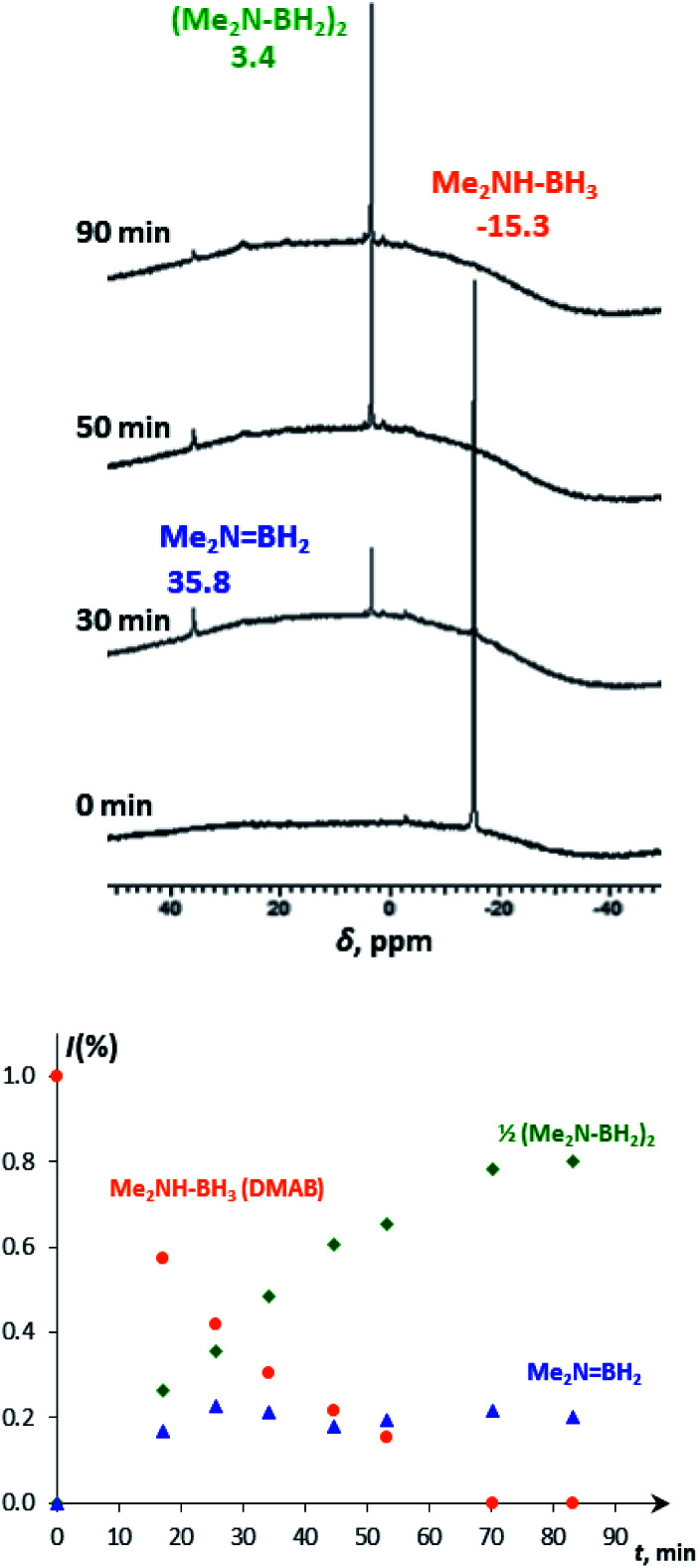
^11^B{^1^H} NMR spectra (128.3 MHz, THF-d_8_) of DMAB (5 equiv.) in the presence of **4a** (top) and the corresponding changes in molar fractions in time (bottom).

At ambient temperature in the presence of up to 50-fold excess of DMAB over **4**, the catalytic dehydrogenation reaction occurs. To deeper understand the mechanism, the reaction was followed spectroscopically and volumetrically. IR monitoring shows the progressive decrease of *ν*_BH_ (2360–2260 cm^−1^) and *ν*_NH_ (3200 cm^−1^) bands of DMAB in the presence of **4a** or **4b** until their complete disappearance (Fig. S6†). This observation provides strong confirmatory evidence for the cleavage of B–H and N–H bonds. A decrease of the typical DMAB signal (*δ*_B_ −15.3) and the accumulation of the dehydrogenation product – *i.e.* the cyclic dimer (Me_2_NBH_2_)_2_ (*δ*_B_ 3.4), as well as the appearance of the steady-state intermediate – aminoborane Me_2_N=BH_2_ (*δ*_B_ 35.8, Fig. 3) – was observed in the ^11^B{^1^H} NMR spectra, evidencing the off-metal dimerization mechanism.^30^

**Fig. 4:**
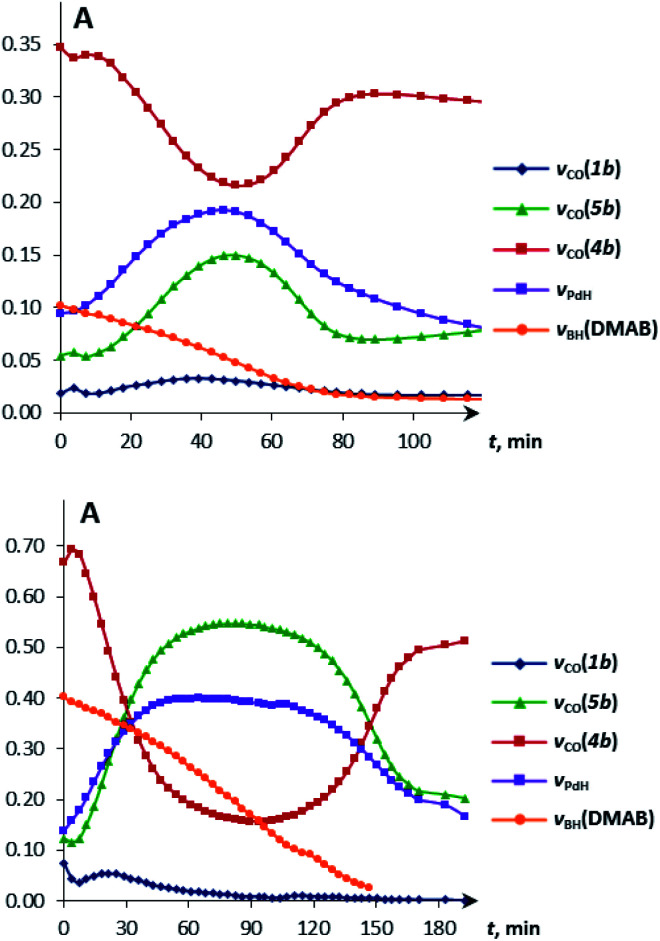
Kinetic curves obtained from the IR spectra of DMAB (1 equiv. (top) and 5 equiv. (bottom)) dehydrogenation catalyzed by **4b** (*c* = 0.003 M). 298 K, THF.

Under these conditions, ^1^H NMR spectroscopy shows the initial appearance of metal hydride resonances (*δ*_WH_ and *δ*_PdH_, Fig. S7 and S8†). Then, the tungsten hydride singlet (*δ*_WH_ −7.4 and −2.3 for **1a** and **1b**, respectively) disappears, but the palladium hydride triplet (*δ*_PdH_ −4.2) still accumulates. ^31^P{^1^H} NMR spectra show the corresponding growth of the (PCP)PdH signal (*δ*_P_ 92.0), whereas the bimetallic complex (*δ*_P_ 75.6) is first consumed and then restored (Fig. S9†). Changes in the IR spectra show the same trend: the ionic complex **5** is formed at the expense of **4**, whereas the *ν*_PdH_ band (1718 cm^−1^) increases in intensity (Fig. S10 and S11†). The bands (*ν*_CO_, *ν*_PdH_) of **4**, **5** and (PCP)PdH restore their initial intensity after the complete conversion of DMAB (Fig. 4, top). An increase in DMAB loading increases the length of the quasi-stationary stage of the reaction when the concentration of both the catalyst and reaction intermediates is nearly constant (Fig. 4, bottom).

**Fig. 5:**
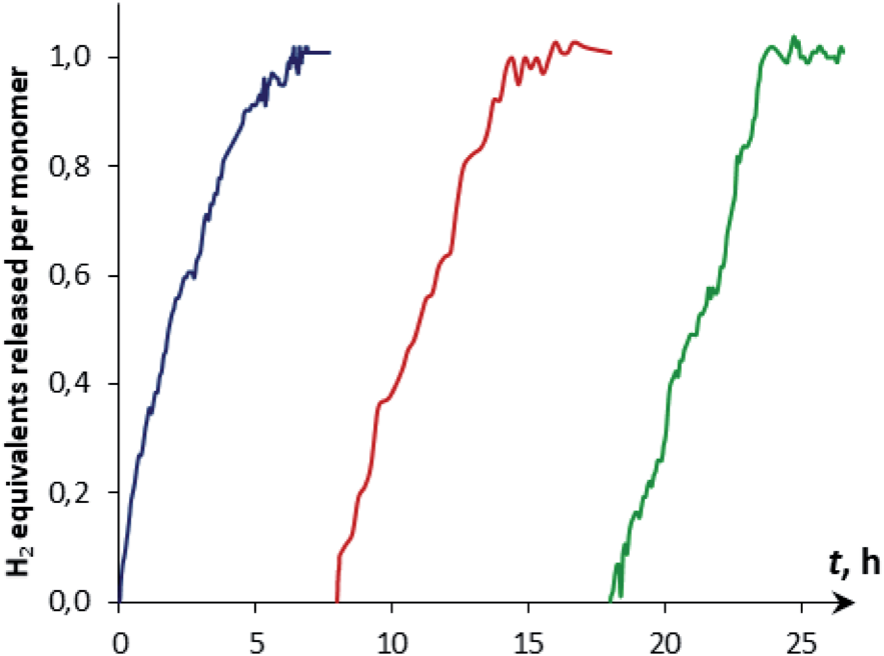
Kinetic curves of DMAB (*c* = 0.06 M) dehydrogenation by reused [CpW(CO)_2_(μ-CO)⋯Pd(PCP)] (**4a**, 5 mol%, *c* = 0.003 M) in THF at 313 K.

The reaction kinetics was measured following the decrease of *ν*_BH_ bond intensity of DMAB. The observed initial rate corresponds to the substrate disappearance (Fig. S6†). In the presence of excess DMAB, the kinetic curves (*c*(DMAB) *vs. t*) are linear (Fig. S12†). DMAB dehydrogenation occurs faster in the presence of the bimetallic complex **4a** with the Cp-ligand: the initial reaction rate *ν*_0_ is 5.8 × 10^−6^ M s^−1^ for **4a** and 1.7 × 10^−6^ M s^−1^ for **4b** at 20 mol% catalyst loading. Keeping the substrate concentration constant, the increase of the catalyst **4a** concentration from 0.003 M to 0.006 M leads to the doubling of the initial reaction rate. It should be noted that the kinetic curves obtained by the integration of proton NMR spectra have very similar behavior to the IR curves (Fig. S13†). According to the spectral data obtained, the bimetallic species [LW(CO)_2_(μ-CO)⋯Pd(PCP)] (**4**) are regenerated at the end of the catalytic reaction and can be reused for the next substrate loading (Fig. 5). The initial reaction rates *ν*_0_ are similar when DMAB is added to the mixture of two neutral hydrides or to the pre-generated bimetallic complex **4**, revealing that the reagents' mixing sequence does not affect the reaction rate (Fig. S11 and S12†).

**Fig. 6:**
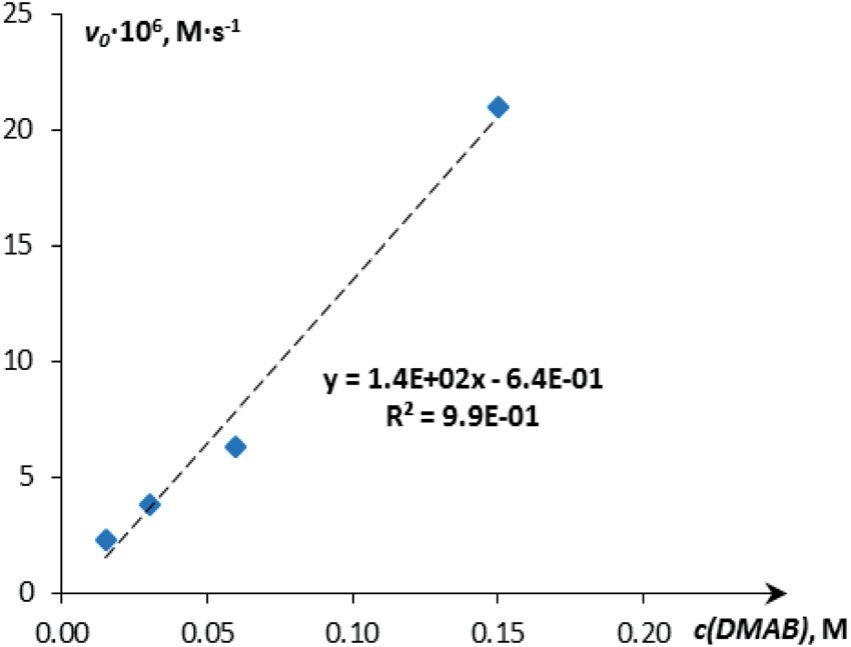
Dependence of the initial reaction rate on the substrate (DMAB) concentration (*c* = 0.015 M, 0.03 M, 0.06 M, 0.15 M; THF; 313 K).

**Fig. 7:**
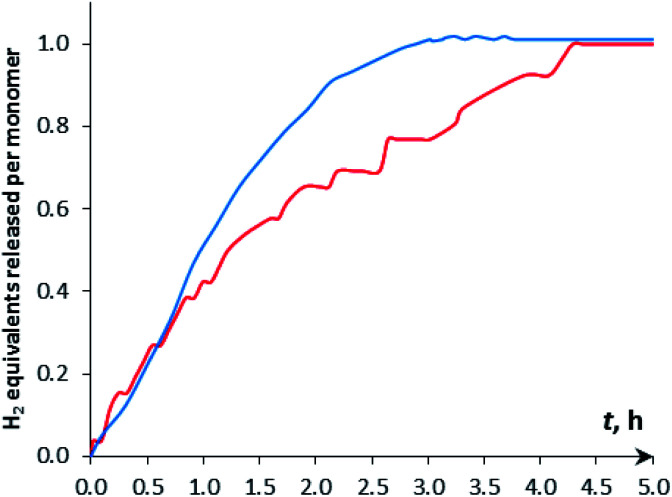
Kinetic curves of DMAB dehydrogenation by [CpW(CO)_2_(μ-CO)⋯Pd(PCP)] (**4a**, *c* = 0.003 M) in THF at 313 K. (Red) *c*(DMAB) = 0.015 M, (5 eq.); (blue) *c*(DMAB) = 0.15 M, (50 eq.).

The H_2_ production in the reaction of DMAB with [CpW(CO)_2_(μ-CO)⋯Pd(PCP)] (**4a**) in THF was monitored using the Man on the Moon X103 kit at ambient temperature and 313 K (Fig. 5 and S14†). At 313 K and 1 : 5 catalyst : DMAB ratio, complete DMAB conversion is achieved in 4 h (Fig. 7 and Table S3†) with an initial reaction rate of 2.5 × 10^−6^ M s^−1^. The increase of substrate loading to 50 equivalents at the same concentration of **4a** (*c* = 0.003 M, 2 mol%; *T* = 313 K) leads to complete DMAB conversion in less than 3 hours (Fig. 7), and the initial reaction rate is 2.1 × 10^−5^ M s^−1^. Under these conditions, the TOF value reaches 26 h^−1^ at a half-conversion time (Table S3†). The initial reaction rate at a catalyst : DMAB ratio of 1 : 50 is higher than that at 1 : 5, since the increase of DMAB amount at a constant catalyst concentration shifts the equilibrium of complex **5** formation (Scheme 2 and Fig. 6).

### Mechanism of R′RNHBH_3_ dehydrogenation by bimetallic ion pairs

**Scheme 3:**
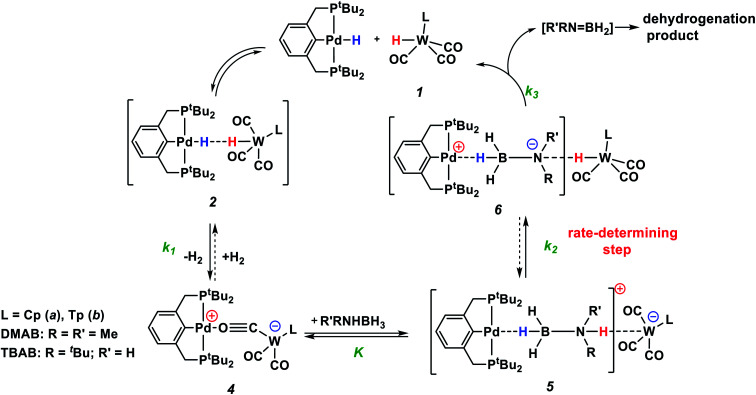
Mechanism of R′RNHBH_3_ dehydrogenation by bimetallic ion pairs **4**.

According to the experimental data, we suggest the reaction mechanism shown in Scheme 3 involving three important steps: (1) amine-borane molecule insertion between the two units of the bimetallic complex, (2) proton and hydride transfer, yielding aminoborane (Me_2_N=BH_2_) and neutral metal hydrides, (3) dihydrogen release as a result of the reaction between palladium and tungsten hydrides *via* the DHB complex. The insertion of the amine-borane molecule into the bimetallic complex **4** yields the η^1^-borane complex **5** (Scheme 3). The latter is also an ionic pair with the tungsten anion [LW(CO)_3_]^−^, which could explain the accumulation of LW(CO)_3_^−^ in the reaction mixture (Fig. 4). In the next stage, the proton transfer from the NH group of coordinated amine-borane to tungsten atom gives the neutral hydride **1** and the zwitterionic complex **6**. The hydride transfer from the BH group to the palladium atom inside complex **6** leads to Me_2_N=BH_2_ elimination and the formation of (PCP)PdH. To close the catalytic cycle, the two neutral metal hydrides react with each other, regenerating the catalytically active complex **4**.

### DFT calculations

**Fig. 8:**
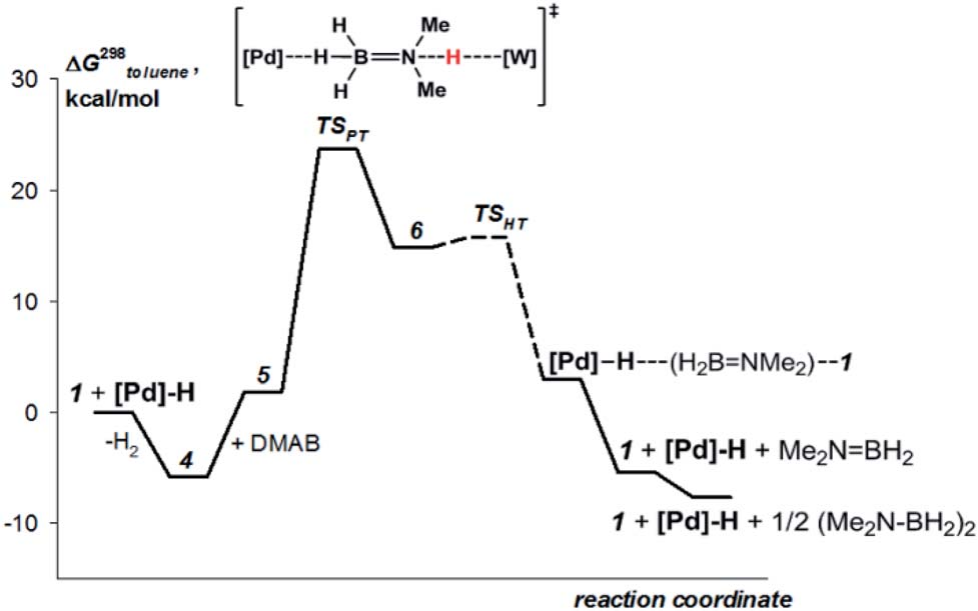
Computed (DFT/ωB97XD) energy profile for the DMAB dehydrogenation by [TpW(CO)_2_(μ-CO)⋯Pd(PCP)] (**4b**) in toluene (SMD model).

**Fig. 9:**
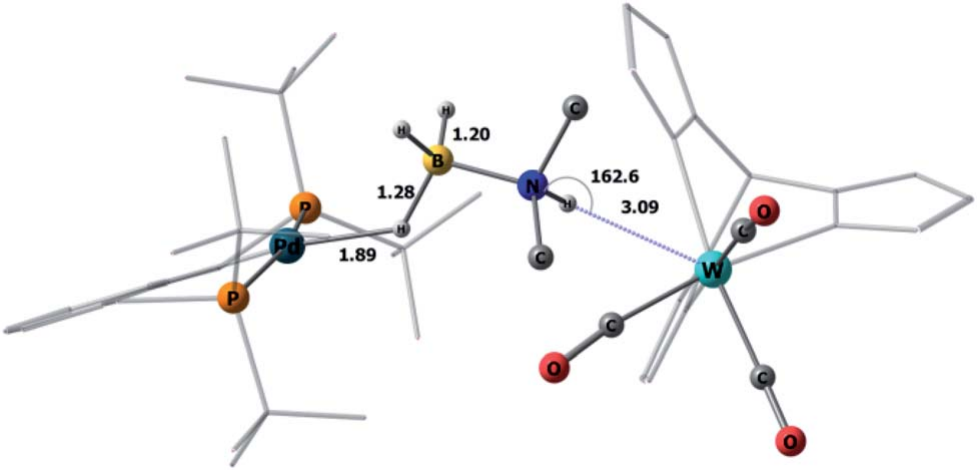
DFT/ωB97XD-optimized structure of the complex **5b**. Key bond lengths are reported in Å.

This mechanism was supported by DFT calculations at the ωB97XD/def2-TZVP theory level taking the real system for the structure optimization in toluene, which was introduced within the SMD model. Complex **5** is 7.7 kcal mol^−1^ above **4** on the Gibbs free energy scale (Fig. 8); however, its formation is possible (electronic energy difference Δ*E*(**4–5**) = −7.5 kcal mol^−1^). The formation of **5** from **1** + **Pd** + DMAB is nearly ergoneutral (Δ*G*(**1–5**) = +1.9 kcal mol^−1^) and thus this equilibrium can be effectively shifted toward **5** in the presence of excess DMAB. The key bonds in complex **5** (Fig. 9) are elongated (*r*(Pd–H_B_) = 1.89 Å and *r*(B–H_Pd_) = 1.28 Å) relative to the B–H bond in free DMAB (1.21 Å) and Pd–H in (PCP)PdH (1.64 Å). The amine group of the dimethylamine fragment is hydrogen-bonded to the tungsten anion (H_N_–W 3.09 Å, ∠N–H–W 162.6°). Despite the B–H_Pd_ bond elongation, the relaxed potential energy surface (PES) scan for BH bond distance in the complex **5** did not lead to any TS, continuously going up to 50 kcal mol^−1^. Hydride transfer does not occur at this step, due to the instability of NR_3_BH_2_^+^, in agreement with the rather low hydride-donating ability of amine-boranes.^31^ This is consistent with the suggested hydride transfer from DMAB to the cationic Pt-catalyst^32^ where additional stabilization of [Me_2_NHBH_2_]^+^ by a strong nucleophile was required. For the DMAB molecule, the proton transfer from nitrogen to tungsten occurs with a barrier Δ*G*^‡^ = 21.8 kcal mol^−1^ and the formation of the zwitterionic complex **6** and LW(CO)_3_H (13.0 kcal mol^−1^ above complex **5**). B–H bond cleavage in complex **6** is almost barrierless (<2 kcal mol^−1^) (Fig. S15†). As a result, the hydride transfer goes faster than the proton transfer (*k*_3_ ≫ *k*_2_); therefore, complex **6** could not be observed experimentally. The ensemble of the reaction products ((PCP)PdH, LW(CO)_3_H and H_2_B=NR_2_) is only slightly higher in free energy than the starting adduct **5** (+1.0 kcal mol^−1^), and the reaction is still reversible. However, since H_2_ evolution from the two hydrides (*k*_1_, Scheme 3)^8,9,33^ and the off-metal B=N dimerization^34,35^ are featured with a comparable barrier, the overall reaction of dehydrogenative DMAB coupling becomes irreversible.

### Catalytic TBAB dehydrogenation

Dehydrogenation of mono-substituted *tert*-butylamine-borane (^*t*^BuNH_2_BH_3_, TBAB) was also studied using complex **4a** as a catalyst. However, in this case, the IR spectroscopic picture and kinetic curves are not similar to those with DMAB (Fig. S16†). On the quasi-stationary stage, the concentration of neutral tungsten hydride **1a** is higher, and that of the η^1^-borane complex **5a** is lower than in DMAB dehydrogenation. Measured 60 min after TBAB addition (*δ*_B_ −21.8), the ^11^B NMR spectrum shows the formation of the intermediate ^*t*^BuNH=BH_2_ (*δ*_B_ 36.1, t) and cyclotriborazane [^*t*^BuNH–BH_2_]_3_ (*δ*_B_ −3.7, t) – the product of the evolution of the first H_2_ equivalent (Fig. S17†). At the end of the reaction, there is no initial TBAB, and the single product of the evolution of the second H_2_ equivalent – borazine [^*t*^BuN–BH]_3_ (*δ*_B_ 28.1, d) – is observed. The formation of two equiv. H_2_ per ^*t*^BuNH_2_BH_3_ molecule was also confirmed by volumetric measurements (Fig. S18†).

To explain the different ratios of ionic and molecular forms of catalyst units in the reaction with DMAB and TBAB, we considered the behavior of the first dehydrogenation product that accumulates in an unexpectedly high amount in the case of TBAB. Monomeric aminoborane H_2_B=NR_2_ is potentially basic^36^ and appears to be able to deprotonate the tungsten hydride (eqn (1)).1
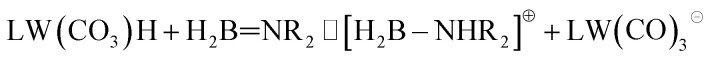


The isolated [H_2_B–NHR_2_]^+^ cation is not stable, but it can be stabilized by interacting with nucleophilic atoms like the carbonyl or the THF's oxygen atoms. Indeed, the neutral CpWH(CO)_3_/H_2_B=NMe_2_ complex appears to be almost ergoneutral relative to the ion pair CpW(CO)_3_^−^/H_2_B–NMe_2_H^+^ (Δ*E* = −1.3 kcal mol^−1^ in favor of the neutral form, Fig. S19†) stabilized by B⋯OC_W_ interactions. Introducing a THF molecule into the system also allows stabilizing the ion pair *via* B⋯O_THF_ interactions (Δ*E* = +0.2 kcal mol^−1^; Fig. S19†), whereas CO groups of the CpW(CO)_3_^−^ anion appear unbounded, having a geometry similar to that of the CpW(CO)_3_^−^/R_3_NH^+^ ion pair.^37^ It should be noted that H_2_B=NH^*t*^Bu appears to be a weaker base than H_2_B=NMe_2_; its neutral form is preferred by 3.8 kcal mol^−1^. That suggests a lower deprotonation extent of LWH(CO)_3_ when H_2_B=NH^*t*^Bu is formed (Fig. S19 and S20†).

Based on these computational data, we could expect the equilibrium side process of LW(CO)_3_H deprotonation, yielding the ion pair CpW(CO)_3_^−^/H_2_B–NMe_2_H^+^ with CO⋯B interaction for DMAB dehydrogenation in toluene. When THF is used as a solvent, this ion pair is likely converted into the THF stabilized one. Consequently, the IR spectra in the *ν*_CO_ range in THF resemble those of the tungsten anion, while the IR spectra in toluene would rather resemble those of the ion pair **4** (Fig. S21†). Thus, this side process (eqn (1)) should affect the solution composition at the quasi-stationary stage of DMAB dehydrogenation, increasing the relative amount of LW(CO)_3_^−^. Switching DMAB to TBAB diminishes the impact of LW(CO)_3_H deprotonation leading to the presence of both metals in hydridic forms in the reaction mixture (Fig. S16†).

### Reaction kinetics

Both DFT calculations and experimental data suggest that the NH-bond cleavage is the rate-determining step of the catalytic reaction. Under these conditions, the overall reaction rate is determined by the rate of proton transfer in complex **5** (**5** → **6**) *r*_2_ = *k*_2_·[**5**]. As shown by DFT calculations, the activation energy values for stepwise proton and hydride transfer (Δ*G*^‡^_PT_ = 21 kcal mol^−1^ and Δ*G*^‡^_HT_ < 2 kcal mol^−1^) indicate that *k*_3_ ≫ *k*_2_ (Scheme 3). Thus, the hydride transfer goes much faster than proton transfer and conversion of **6** to products has no influence on the reaction rate. When DMAB is in excess, the reaction is pseudo-zero order in DMAB. Taking into account the pre-kinetic step of DMAB coordination to bimetallic complex **4** in the presence of excess DMAB, the reaction rate is *r* = *k*_2_*Kc*_0_(DMAB)·[**4**] **=***k*_eff_·[**4**], *K* = [**5**]/([**4**][DMAB]). Thus, analysis of the experimental data gives the *k*_2_ values of the rate-limiting step equal to 0.17 s^−1^ and 0.03 s^−1^ for **4a** and **4b**, respectively (Table S4†). These values correspond to Δ*G*^‡^_298 K_ 18.5 ± 0.1 and 19.5 ± 0.1 kcal mol^−1^, in reasonable agreement with DFT calculations. For TBAB dehydrogenation by complex **4a**, the *k*_2_ value is 0.10 s^−1^ (Δ*G*^‡^_298 K_ 18.8 ± 0.1 kcal mol^−1^), indicating faster proton transfer. The overall activation free energy Δ*G*^‡^_298 K_ for the conversion of **4** to **6** is *ca.* 25 kcal mol^−1^ that is in good agreement with DFT calculations (Fig. 8).

The use of deuterated amine-boranes NDMe_2_BH_3_ and NDMe_2_BD_3_ proves that the rate-limiting stage is the N–H bond cleavage, as is often for AB dehydrogenation.^38^ The rate constant *k*_eff_ for NDMe_2_BH_3_ dehydrogenation catalyzed by **4a** (Fig. S22 and S23†) is substantially lower (*k*^ND^_eff_ = 2.8 × 10^−7^ M s^−1^) than that for DMAB (*k*^NH^_eff_ = 5.8 × 10^−6^ M s^−1^) giving the kinetic isotope effect (KIE = *k*^NH^/*k*^ND^) of 20.6 ± 0.3. The use of the fully deuterated analogue NDMe_2_BD_3_ does not lead to a further decrease in the reaction rate, giving KIE = *k*^H^/*k*^D^ of 20.6 ± 0.7. The KIE obtained is substantially higher than typical KIEs in metal-catalyzed amine-borane dehydrogenation.^39–43^ This value also exceeds the KIEs for self-exchange in CpW(CO)_3_H/[CpW(CO)_3_]^−^ or for proton transfer of CpW(CO)_3_H to aniline.^44,45^ Such high KIE values suggest a proton tunneling that is likely to occur when there is a minimal geometry distortion along the reaction coordinate.^46^ Our computations partially reproduce the observed magnitude of the isotope effect predicting increasing of the barrier upon NH to ND substitution by ΔΔ*G*^‡^_298_ = 1.5 kcal mol^−1^ (KIE = *k*^NH^/*k*^ND^ = 13). One of the reasons for large KIE magnitudes (>10) is long (large acid–base separations) and strong H-bonded complexes.^47^

## Conclusions

In summary, bimetallic complexes [LW(CO)_2_(μ-CO)⋯Pd(PCP)] (**4**) act as “metallic-analogs” of typical main group FLPs. The presence of acidic and basic metal centers in these ionic pairs triggers the cooperative BH/NH bond activation in amine-boranes. In the first reaction stage, the η^1^-borane complex [(PCP)Pd-(σ^1^-HBH_2_–NR_2_H)⋯W(CO)_3_L] (**5**) is formed, in which BH is strongly bound to the palladium atom and the amine group is hydrogen-bonded to the tungsten atom. The step-wise proton transfer to W and hydride transfer to Pd yield the unsaturated B=N fragment and neutral metal hydrides. Molecular hydrogen evolution is the result of two metal hydrides interacting, regenerating bimetallic species **4**. One equivalent of H_2_ can be produced from Me_2_NHBH_3_, whereas dehydrogenation of ^*t*^BuNH_2_BH_3_ gives two equivalents of hydrogen per monomer at room temperature. The catalytic system can be easily generated through direct mixing of LWH(CO)_3_, (PCP)PdH and amine-borane, without preliminary synthesis of the ionic catalyst **4**. The presence of ionic intermediates during the dehydrogenation cycle requires the use of polar solvents to achieve effective catalysis. The mechanistic study showed that proton transfer is the rate-determining step; therefore, the reaction is accelerated by a more basic anion ([CpW(CO)_3_]^−^ > [TpW(CO)_3_]^−^) while the hydride transfer is almost barrierless. The dehydrogenation process starts only in the presence of excess amine-borane due to the shift of the pre-kinetic equilibrium (H_3_BNHR_2_ + **4** = **5**) to the right, which in turn causes the increase of the initial reaction rate. Yet another notable observation is that the dimethylaminoborane monomer H_2_B=NR_2_ is able to deprotonate tungsten hydride in competition with H_2_ evolution from two hydrides and H_2_B=NR_2_ oligomerization. Different basicities of dehydrogenated DMAB and TBAB monomers lead to a different impact of this side process to the overall catalytic reaction.

Prior studies have shown that bimetallic systems featuring a metal–metal bond^48^ can indeed operate in a concerted way activating H_2_, C–H, B–H, and other bonds.^49–52^ These complexes activate the ONE bond, splitting it between two metals, often in an oxidative addition fashion. In our case, two transition metal-based building units of a bimetallic complex do not interact directly but act cooperatively as a Lewis acid and a Lewis base, splitting the N–H and B–H bond without changing the metals' oxidation state. So far, such reactivity has been reported only for [Cp_2_ZrOC_6_H_4_P^*t*^Bu_2_]^+^ which can be described as an early transition-metal-containing linked FLP.^21^ This behavior is similar to that of Stephan's main group FLPs^18^ and can be exploited for other catalytic conversions; these studies are underway in our laboratories.

## Experimental section

All reactions were performed using standard Schlenk procedures under a dry argon atmosphere. Commercial reagents (dimethylamine-borane, *tert*-butylamine-borane) were purchased from Aldrich and used after preliminary sublimation. Tetrahydrofuran (THF) and toluene were dried over Na/benzophenone and distilled under an argon atmosphere. THF-d_8_ (Aldrich) was stored over 4 Å molecular sieves and degassed before use by three freeze–pump–thaw cycles. NDMe_2_BH_3_ and NDMe_2_BD_3_ were prepared as described in the literature.^40,53^ Variable-temperature (VT) NMR spectra were recorded on Bruker AVANCE II and Varian Inova FT-NMR spectrometers operating at 300 and 400 MHz in the 200–320 K temperature range. ^1^H chemical shifts are reported in parts per million (ppm) downfield of tetramethylsilane (TMS) and were calibrated against the residual resonance of the deuterated solvent, while ^31^P{^1^H} chemical shifts were referenced to 85% H_3_PO_4_ with downfield shift taken as positive. ^11^B and ^11^B{^1^H} were referenced to BF_3_·OEt_2_. IR spectra were recorded at different temperatures (190–293 K) using a home-modified cryostat (Carl Zeiss Jena) with a Nicolet 6700 spectrometer using 0.05–0.2 cm CaF_2_ cells. The accuracy of the experimental temperature was ±0.5 °C. The cryostat modification allows transferring the reagents (premixed at either low or room temperature) under an inert atmosphere directly into the cells.

Elemental analyses were carried out in the Laboratory of Microanalysis of INEOS RAS. The classic manual technique was used. The sample was burned in a platinum crucible in a stream of oxygen at 950 °C followed by trapping CO_2_ and water with Ascaris (asbestos impregnated with NaOH) and Anhydrone (anhydrous magnesium perchlorate), respectively, and the analysis of the mass changes.

### Synthesis of [LW(CO)_2_(μ-CO)⋯Pd(PCP)] L = Cp (**4a**), Tp (**4b**)

Solid LW(CO)_3_H (0.03 mmol) was placed in a Schlenk flask filled with argon (10 ml) together with 1 ml of tetrahydrofuran. Then, 1 ml of a (PCP)PdH THF solution (0.03 mmol of hydride in 2 ml of the solvent) was added to this solution. The resulting mixture of two colorless hydrides instantly became yellow colored. After an hour of stirring, the solvent was concentrated to *ca.* 0.1 ml under vacuum. Then, the Schlenk flask was filled with argon and left for several days standing at ambient temperature until a crystalline precipitate was obtained. The resulting solid (yellow needle-shaped crystals) was washed with a small amount of cold THF (2 × 0.2 ml) and dried in a vacuum. Yield: 90%.

#### 4a

IR (THF, *ν*_CO_, cm^−1^): 1907, 1815, 1656. ^1^H̲ NMR (400 MHz, THF-d_8_, ppm): *δ* 6.93–6.85 (m, 3H, ArH); 5.10 (s, 5H, Cp–H), 3.28 (t, ^2^*J*_P–H_ = 4.12 Hz, 4H, –CH_2_–); 1.43 (t, ^3^*J*_P–H_ = 7.0 Hz; 36H, C(CH_3_)_3_). ^31^P{^1^H} (161.9 MHz, THF-d_8_, ppm): *δ* 75.3. Anal. calc. (%) for C_32_H_47_O_3_P_2_PdW (%): C 46.20, H 5.69. Found: C, 46.00; H, 5.78.

#### 4b

IR (THF, *ν*_CO_, cm^−1^): 1901, 1796, 1650. ^1^H̲ NMR (600 MHz, THF-d_8_, ppm): *δ* 7.66 (s, 3H, Tp CH), 7.59 (s, 3H, Tp CH), 5.98 (s, 3H, Tp CH), 6.82–6.76 (m, 3H, ArH), 3.19 (pt, ^2^*J*_P–H_ = 4.12 Hz, 4H, –CH_2_–), 1.24 (pt, ^3^*J*_P–H_ = 7.0 Hz, 36H, C(CH_3_)_3_). ^31^P{^1^H} (161.9 MHz, THF-d_8_, ppm): *δ* 76.8. Anal. calc. (%) for C_36_H_53_BN_6_O_3_P_2_PdW (%): C 44.08, H 5.45. Found: C, 43.95; H, 5.53.

### General procedure for DMAB dehydrogenation by **4**

#### For NMR kinetic studies

(PCP)PdH (*c* = 0.01 M) and **1a** or **1b** complexes (*c* = 0.01 M) and DMAB (5, 10 eq.) were mixed simultaneously at 290 K in THF-d_8_.

#### For variable temperature IR studies

Complex **4a** or **4b** was generated *in situ* by the reaction of (PCP)PdH (*c* = 0.003–0.0039 M) with **1a** or **1b** (*c* = 0.003 M), respectively. The reagents were dissolved in THF or toluene at 270 K and allowed to react for 30 min. Then, a chosen amount of DMAB was added, and the obtained mixture was monitored in the temperature range 190–290 K.

#### For kinetic IR studies

Reagents were prepared *via* three methods.

##### Method I

A portion of isolated complex **4a** or **4b** was dissolved in THF (*c* = 0.003 M). Then, a chosen amount of DMAB (5 equiv.) was added and the IR spectra were monitored until full catalyst regeneration.

##### Method II

Complex **4a** or **4b** was generated *in situ* by the reaction of (PCP)PdH (*c* = 0.003–0.0036 M) with **1a** or **1b** (*c* = 0.003 M), respectively. The reagents were dissolved in THF at 290 K and allowed to react for 20 min. Then, a chosen amount of DMAB (1–5 eq.) was added, and the mixture obtained was monitored until full catalyst regeneration.

##### Method III

(PCP)PdH (*c* = 0.003–0.0036 M), **1a** or **1b** complexes (*c* = 0.003 M) and DMAB (5 eq.) were mixed simultaneously at 290 K in THF. The reaction mixture was monitored until full catalyst regeneration.

#### Volumetric studies of amine-borane dehydrogenation

Hydrogen evolution during dehydrogenation of amine-boranes was monitored using the Man on the Moon X103 kit. The volume of the system is 32 ml (two-necked round-bottom flask – 30 ml, three-way valve – 2 ml). The monitored solutions were prepared *via* three methods.

##### Method I

A portion of the isolated complex **4a** or **4b** (0.006 mmol) was dissolved in THF (1 ml) in an argon-filled flask of the device connected to a three-way valve. Then the flask was tightly closed with a septum cap, and the valve was opened to the pressure sensor. The chosen amount of DMAB (5–50 equiv.) in 1 ml of THF was injected with a syringe through a septum cap.

##### Method II

Complex **4a** or **4b** (*c* = 0.003–0.01 M) was generated *in situ* by mixing the solutions of (PCP)PdH (1–1.2 eq.) and **1** (*c* = 0.003–0.01 M, 1 eq.) in THF in an argon-filled flask of the device connected to a three-way valve. Then, the flask was tightly closed with a septum cap, and the valve was opened to the pressure sensor. A chosen amount of DMAB (5–50 eq.) in THF was injected with a syringe through a septum cap.

##### Method III

The solution of (PCP)PdH in THF (1–1.2 eq.) was prepared in an argon-filled flask of the device connected to a three-way valve. Then the flask was tightly closed with a septum cap, and the valve was opened to the pressure sensor. The mixture of **1** (*c* = 0.003–0.01 M, 1 eq.) and amine-borane (5–50 equiv.) in THF was injected with a syringe.

The resulting mixture was stirred at 295 K. Data from a pressure sensor connected *via* a wireless network to a computer were recorded as a function of pressure *versus* time for 3–20 hours. The values accumulated were referenced by the pressure of THF in a blank experiment at 295 K and used for calculations of the H_2_ equivalents evolved. The calculations were performed in the ideal gas approximation (*pV* = *nRT*).

### Computational details

Calculations were performed with the Gaussian 09 (ref. 54) package at the DFT/ωB97XD^55^ level without any ligand simplification. For all atoms, the Def2-TZVP^56^ basis set was applied, supplemented with an effective core potential^57^ in the case of Pd and W. The structures of all complexes and transition states were fully optimized in toluene (*ε*_r_ = 2.3741) described by the SMD model,^58^ without any symmetry restrictions. The nature of all the stationary points on the potential energy surfaces was confirmed by vibrational analysis. Transition state (TS) structures showed only one negative eigenvalue in their diagonalized force constant matrices, and their associated eigenvectors were confirmed to correspond to the motion along the reaction coordinate under consideration using the Intrinsic Reaction Coordinate (IRC) method.^59^

### X-ray crystallography

For crystals of **4a** and **4b**, X-ray diffraction data were collected at 120 K with a Bruker ApexII DUO diffractometer using graphite monochromatic Cu-Kα and Mo-Kα radiation, respectively. Using Olex2,^60^ the structures were solved with the ShelXT^61^ structure solution program using intrinsic phasing and refined with the XL^62^ refinement package using least-squares minimization. Hydrogen atom of the BH group in **4b** was found in the difference Fourier synthesis while positions of other hydrogen atoms were calculated, and they all were refined in the isotropic approximation within the riding model. Crystallographic data and structure refinement parameters are given in Table S1.† CCDC 2020560 and 2020559 contain the supplementary crystallographic data for **4a** and **4b**, respectively.†

## Conflicts of interest

There are no conflicts to declare.

## Supplementary Material

SC-012-D0SC06114J-s001

SC-012-D0SC06114J-s002
