# Supplementary material for: Bifunctional activation of amine-boranes by the W/Pd bimetallic analogs of “frustrated Lewis pairs”
Source: Chem Sci. 2021 Jan 15;12(10):3682–92. doi: 10.1039/d0sc06114j (PMC8179527; doi:10.1039/d0sc06114j)
Supplement: SC-012-D0SC06114J-s001 [file SC-012-D0SC06114J-s001.pdf]

Electronic Supplementary Material (ESI) for Chemical Science.
This journal is © The Royal Society of Chemistry 2021

Bifunctional activation of amine-boranes by the W/Pd bimetallic analogs of "frustrated Lewis pairs"

Elena S. Osipova[a], Ekaterina S. Gulyaeva[a], Evgenii I. Gutsul[a], Vladislava A. Kirkina[a], Alexander A. Pavlov[a], Yulia V. Nelyubina[a], Andrea Rossin[b], Maurizio Peruzzini[b],* Lina M. Epstein[a], Natalia V. Belkova[a],* Oleg A. Filippov[a], Elena S. Shubina[a]*

[a] A.N.Nesmeyanov Institute of Organoelement Compounds, Russian Academy of Sciences (INEOS RAS), Vavilova Str. 28, 119991 Moscow, Russia

[b] Istituto di Chimica dei Composti Organometallici – Consiglio Nazionale delle Ricerche (ICCOM - CNR), Via Madonna del Piano 10, 50019, Sesto Fiorentino, Italy.

Supporting Information

Figure S1. IR spectra of ***1b*** (*c* = 0.003 M) and (PCP)PdH (*c* = 0.0045 M) mixture. THF, 190-290 K. 6
Figure S2. IR spectra of ***1b*** and (PCP)PdH mixture in fluorobenzene. 7
Figure S3. IR spectra of ***4b*** (*c* = 0.003 M, *T* = 270 K) and after DMAB addition (*c* = 0.01 M), 270, 230–190 K. *l* = 2 mm, THF....7
Figure S4. IR spectra of ***4a*** (*c*= 0.003 M, red) and after addition of 1eq. DMAB (blue). 190 K, *l* = 0.4 mm, *T* = 190K, toluene. 8
Figure S5. Diagram showing the temperature dependence of ν_{PdH} and ν_{BH} bands intensity in the mixture of ***4b*** (*c* = 0.01 M) and DMAB (*c* = 0.033 M) in toluene. 8
Figure S6. Time evolution IR spectra of ***4a*** and DMAB mixture (1:5) in THF at 298K. 9
Figure S7. Time evolution ^{1}H NMR spectra (400 MHz, hydride region) of ***4a*** in the presence of excess DMAB (5 equiv). 290 K, THF-d_8. 9
Figure S8. Time evolution of ^{1}H NMR spectra (400 MHz, hydride region) of ***4b*** in the presence of excess DMAB (5 equiv.). 290 K, THF-d_8. 9
Figure S9. ^{31}P NMR spectra (161.98 MHz) of ***4a*** in the presence of excess DMAB (5 equiv.), monitoring in THF-d_8 at 290 K. 10
Figure S10. Time evolution of IR spectra of complex ***4b*** (*c* = 0.01 M) with DMAB (*c* = 0.033 M), 298 K, l = 2 mm, THF. 10
Figure S11. Kinetic curves obtained from IR spectra of dehydrogenation reaction of DMAB (5 equiv.) by ***4a*** at 298 K in THF. (a) Addition of DMAB to pregenerated ionic pair ***4a*** (*c* = 0.003 M); (b) simultaneous addition of DMAB, ***1a*** and (PCP)PdH (1:1, *c* = 0.005 M). 11
Figure S12. *c*(*t*) plots of DMAB dehydrogenation by ***4a*** (*c* = 0.003 M, red and blue) and upon the simultaneous mixing of three reagents (***1a*** + (PCP)PdH, 1:1, *c* = 0.005 M, green). 11
Figure S13. Normalized kinetic curves obtained from ^{1}H NMR spectra of dehydrogenation reaction of DMAB (5 eq.) by ***4a*** (*c* = 0.01 M), 293 K, THF-d_8. 11
Figure S14. "Man of the moon" device during a catalytic run. 12
Figure S15. Relaxed PES scan for hydride transfer in complex ***6b***. $TpWH(CO)_3$ and hydrogen atoms, except BH/PdH are omitted. 13
Figure S16. Kinetic curves obtained from IR spectra of dehydrogenation reaction of TBAB (5 eq.) by ***4a*** (*c* = 0.003 M), 298 K, THF. 13

Figure S17. ^{11}B NMR spectrum (128.3 MHz) of TBAB (5 equiv.) in presence of ***4a*** an hour after mixing. 14
Figure S18. Kinetic curve of TBAB dehydrogenation by [CpW(CO)$_2$(μ-CO)···Pd(PCP)] (***4a***, 20 mol.%, *c* = 0.003 M) in THF at 296 K. 14
Figure S19. Optimized structures of neutral (right) and ionic (left) complexes of CpW(CO)$_3$H with H_2BNR_2 and stabilized by THF. The energies are in kcal/mol respective to the neutral pair. 15
Figure S20. Optimized structures of neutral (right) and ionic (left) complexes of TpW(CO)$_3$H with H_2BNR_2. The energies are in kcal/mol respective to the neutral pair. 16
Figure S21. IR spectra of ***4b*** and DMAB mixture (1:1, *c*= 0.003 M) in THF and toluene, 190 K, *l* = 0.5 mm. 17
Figure S22. Kinetic curves obtained from IR spectra of dehydrogenation reaction of Me_2NDBH_3 (5 equiv.) by ***4a*** (*c* = 0.003 M), 298 K, THF. Initial (a) and full regions (b). 17
Figure S23. *c(t)* plots obtained from IR spectra of 5 equiv. Me_2NDBH_3, Me_2NDBH_3 and Me_2NHBH_3 dehydrogenation by **4a** (c = 0.003 M), 298K, THF 18

Table S1. Crystallographic data and structure refinement parameters for ***4a*** and ***4b***. 3
Table S2. Selected structural parameters for complexes [LW(CO)$_2$(μ-CO)···Pd(PCP)] (L = Cp (***4a***), Tp (***4b***)). 4
Table S3. The catalytic performance of ***4a*** in the dehydrogenation of DMAB. 12
Table S4. Experimental rate constants for Me_2NHBH_3 (DMAB) and $^tBuNH_2BH_3$ (TBAB) dehydrogenation and corresponding activation free energies $\Delta G^{\neq}$ (kcal/mol) 19
Kinetic analysis 18
References 19

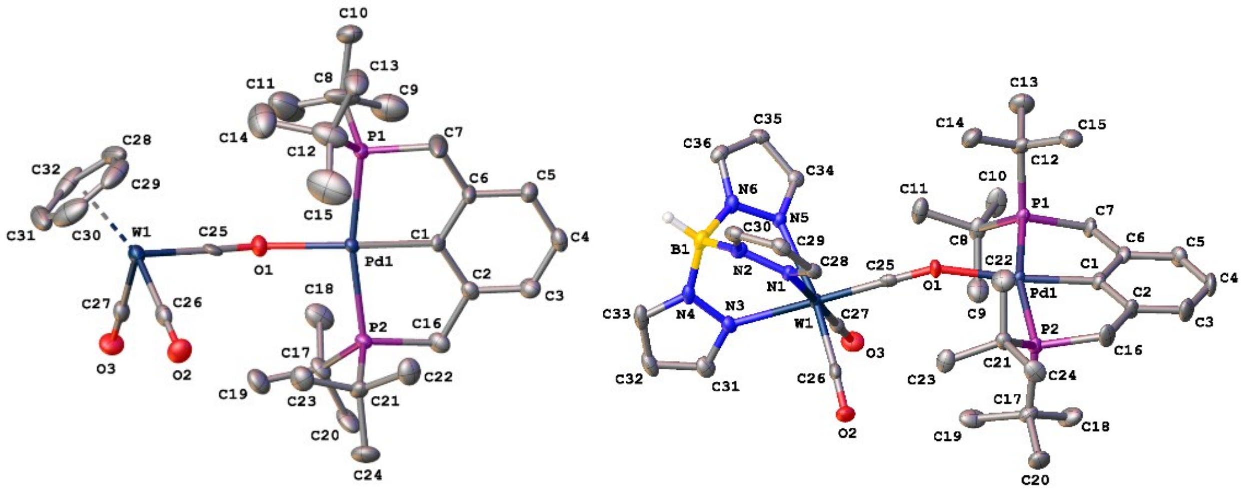

Table S1. Crystallographic data and structure refinement parameters for ***4a*** and ***4b***.

Compound	***4a***	***4b***
Empirical formula	$C_{32}H_{48}O_3P_2PdW$	$C_{36}H_{53}BN_6O_3P_2PdW$
Formula weight	832.89	980.84
Crystal system	Monoclinic	Monoclinic
Space group	$P2_1/c$	$P2_1/c$
a (Å)	8.2012(2)	16.8853(7)
b (Å)	37.3720(8)	15.2569(7)
c (Å)	10.9355(2)	15.7122(8)
β (°)	103.3880(10)	91.7010(10)
V (Å^3)	3260.59(12)	4046.0(3)
D_{calc} (g cm^{-3})	1.697	1.610
Linear absorption, μ (cm^{-3})	120.32	34.06
F(000)	1656	1960
$2\theta_{max}$, °	135	56
Reflections measured	45938	30841
Independent reflections (R_{int})	5834 (0.1108)	9756 (0.0704)
Observed reflections ($I > 2\sigma(I)$)	4578	7383
Parameters	365	463
R_1 (on F for obs. refls)	0.0427	0.0386
wR_2 (on F^2 for all refls)	0.1000	0.0813
Goodness-of-fit	1.030	0.984
Largest diff. peak and hole (e Å^{-3})	1.702/-0.838	1.446/-0.976

Table S2. Selected structural parameters for complexes [LW(CO)$_2$(μ-CO)···Pd(PCP)] (L = Cp (***4a***), Tp (***4b***)).

**Complex**		***4a***	***4b***
Distances, Å	Pd-C(*1*)	2.006(6)	2.009(5)
	Pd-O(*1*)	2.149(4)	2.156(3)
	Pd-P(*1*)	2.311(2)	2.312(1)
	Pd-P(*2*)	2.304(2)	2.316(1)
	W-C(*25*)	1.906(7)	1.899(5)
	W-C(*26*)	1.955(8)	1.944(5)
	W-C(*27*)	1.927(7)	1.959(5)
	C(*25*)-O(*1*)	1.194(8)	1.218(6)
	C(*26*)-O(*2*)	1.16(1)	1.172(6)
	C(*27*)-O(*3*)	1.177(9)	1.174(6)
Angles, °	Pd-O(*1*)-C(*25*)	151.3(5)	151.9(3)
	W-C(*25*)-O(*1*)	176.3(6)	179.0(4)
	C(*1*)-Pd-O(*1*)	174.0(2)	173.2(2)
	P(*1*)-Pd-P(*2*)	167.38(6)	166.47(5)
	P(*1*)-Pd-O(*1*)	96.6(1)	96.78(9)
	P(*2*)-Pd-O(*1*)	95.7(1)	96.54(9)

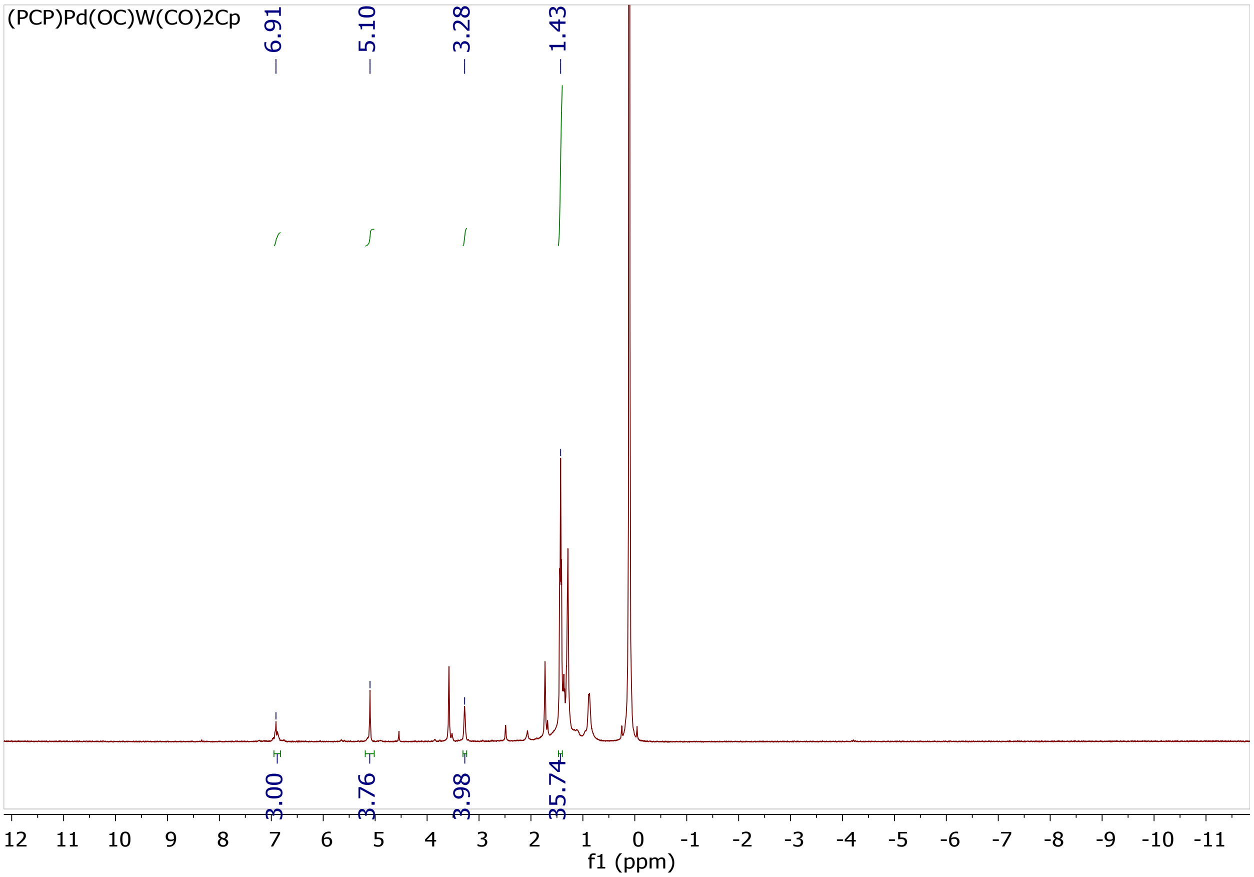

^{1}H NMR spectrum (400 MHz) of complex ***4a***. 290K, THF-*d*^{8}.

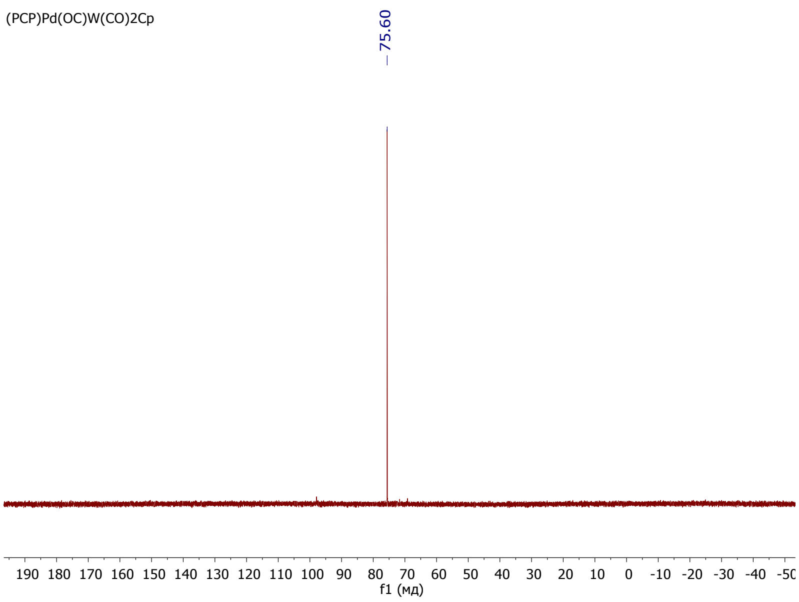

$^{31}P\{^1H\}$ NMR spectrum (161.9 MHz) of complex ***4a***. 290K, THF-*d*^{8}.

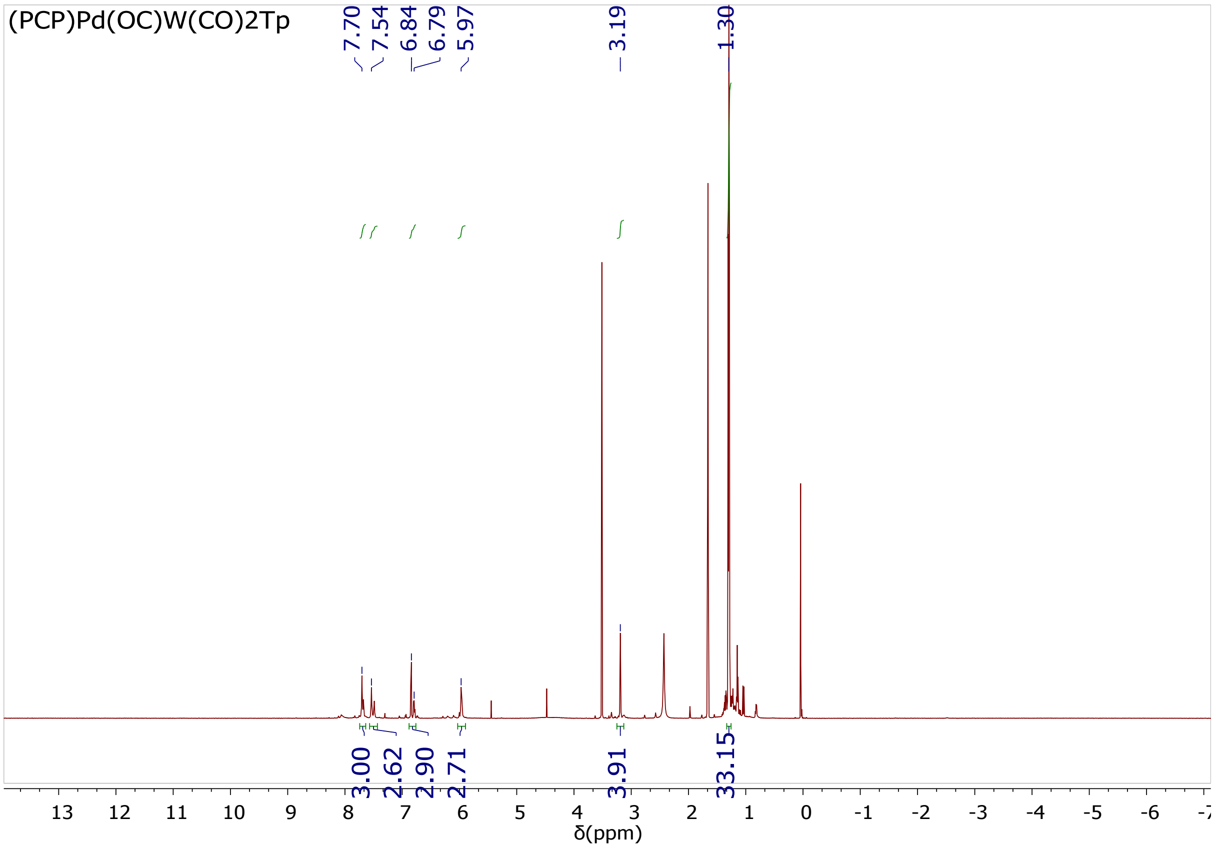

^1H NMR spectrum (600 MHz) of complex ***4b***. 290K, THF-*d*^{8}.

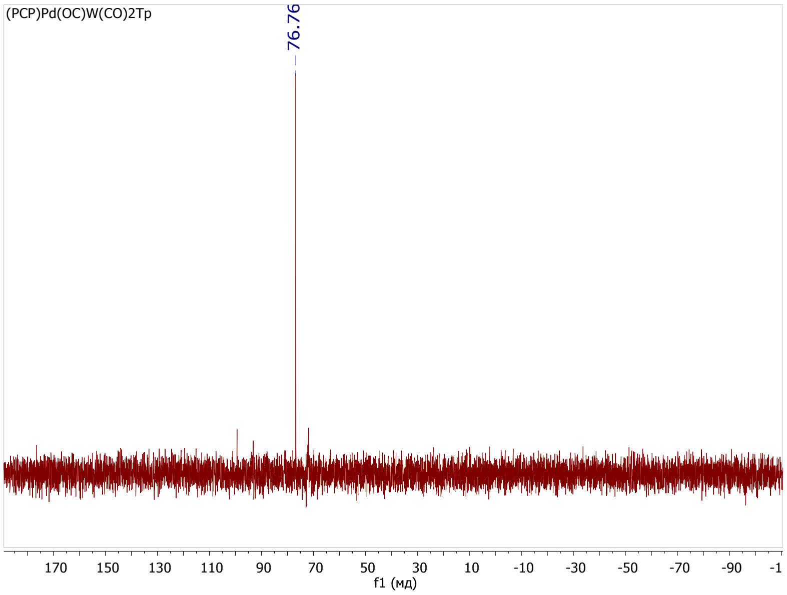

$^{31}P\{^1H\}$ NMR spectrum (161.9 MHz) of complex ***4b***. 290K, THF-d^8.

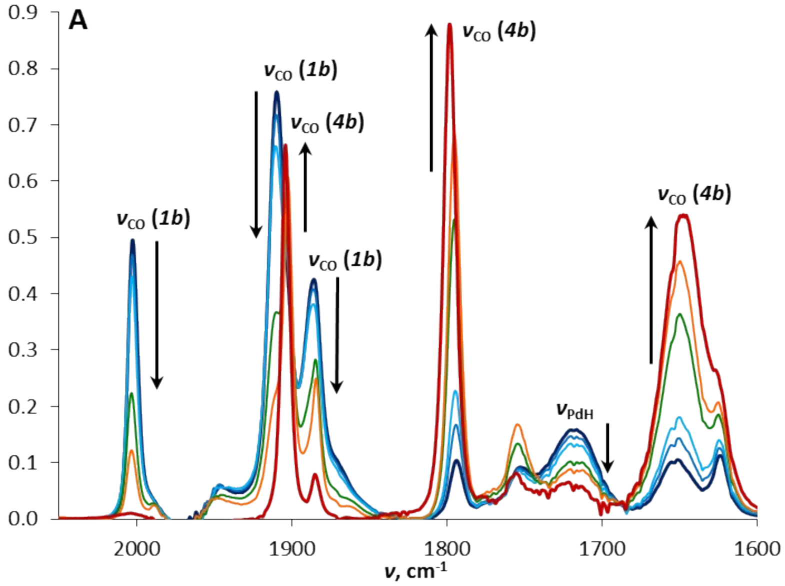

Figure S1. IR spectra of ***1b*** (c = 0.003 M) and (PCP)PdH (c = 0.0045 M) mixture. THF, 190-290 K.

Reversibility of H_2 loss

When fluorobenzene used as a solvent the spectroscopic picture (see Figure 1, main text) is similar to that observed in THF. If the reaction mixture is kept for one day in a closed system after all hydrogen is released at 270-290 K, a band redistribution is observed in the spectrum (Figure S4). Further cooling to 240K leads to significant growth of ν_{CO} of intermediate ***3*** and complex ***4*** and ***1*** ν_{CO} bands intensity decrease. Repeated warming returns the system to the same form, confirming the existence of dynamic equilibrium (1).

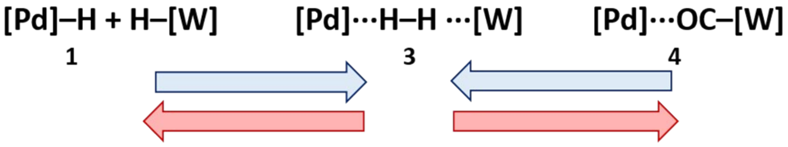

(1)

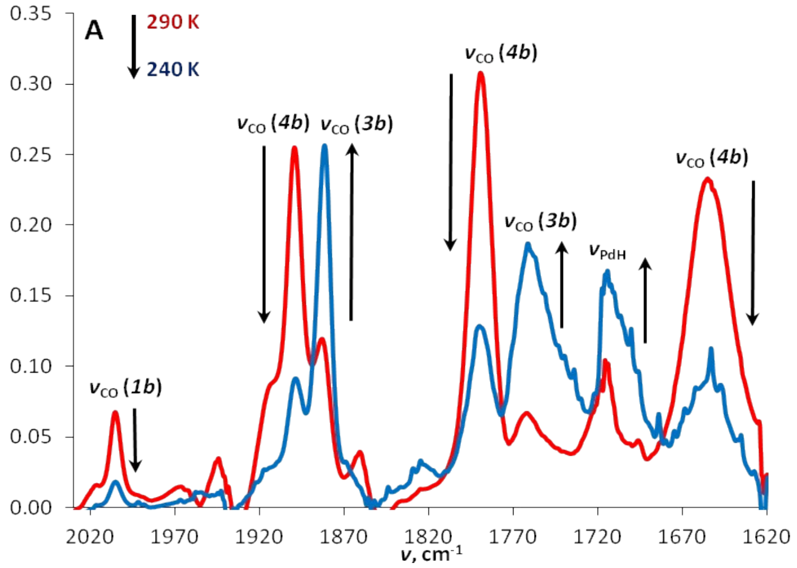

Figure S2. IR spectra of ***1b*** and (PCP)PdH mixture in fluorobenzene.

The rate of H_2 evolution in bimetallic system (***1b*** + (PCP)PdH) increases with the temperature increase from 220 K to 240 K. However, the change is too small in this temperature range to give reliable $\Delta H^{\neq}$ and $\Delta S^{\neq}$ values. [220K: k_{obs} = 0.159 mol^{-1}·s^{-1}, $\Delta G^{\neq}$ = 13.5 kcal/mol; 230K: k_{obs} = 0.178 mol^{-1}·s^{-1}, $\Delta G^{\neq}$ = 14.1 kcal/mol; 240K: k_{obs} = 0.202 mol^{-1}·s^{-1}, $\Delta G^{\neq}$ = 14.7 kcal/mol in THF].

DMAB dehydrogenation

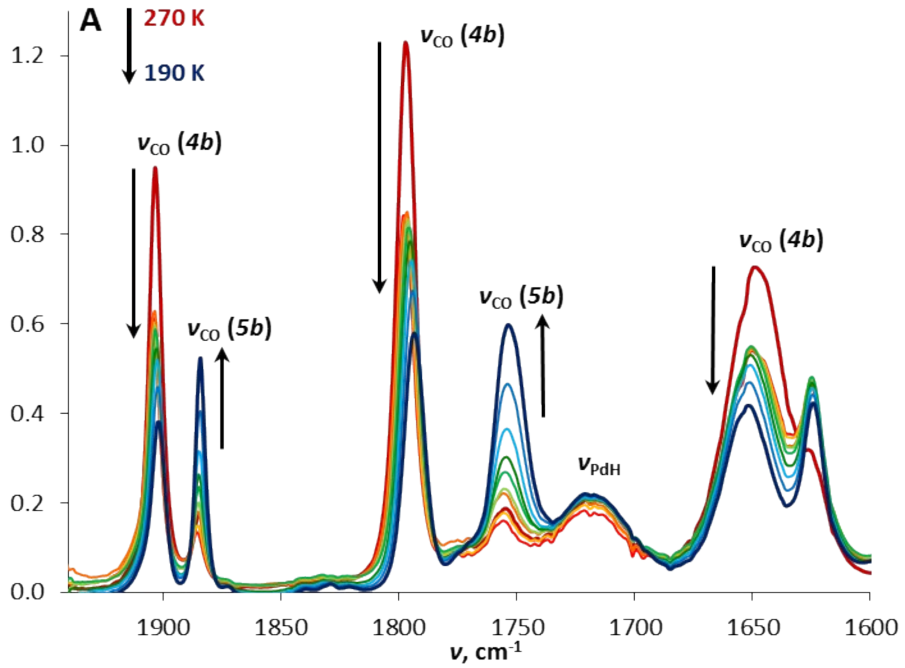

Figure S3. IR spectra of ***4b*** (c = 0.003 M, T = 270 K) and after DMAB addition (c = 0.01 M), 270, 230–190 K. l = 2 mm, THF.

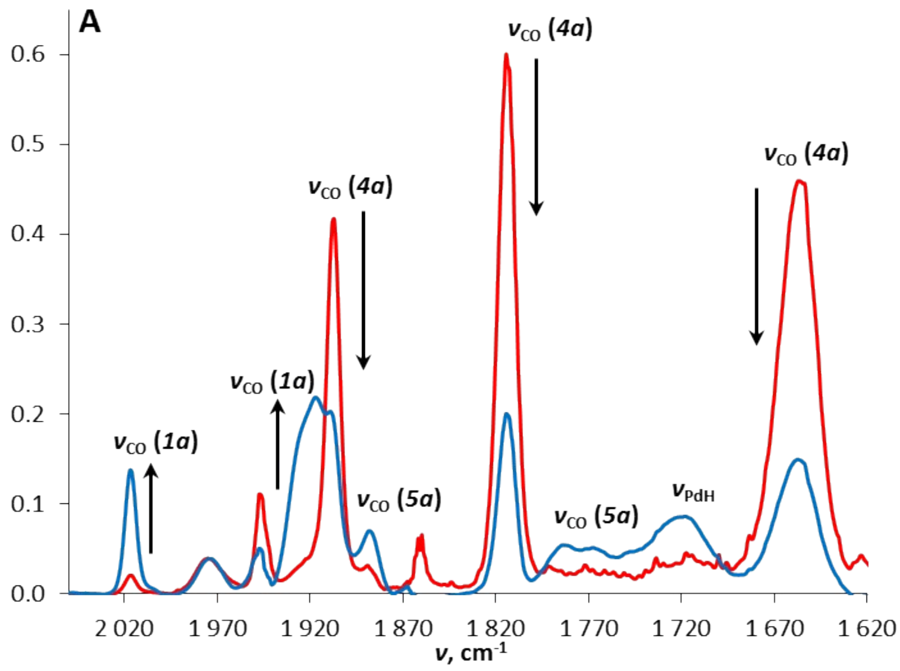

Figure S4. IR spectra of ***4a*** (*c*= 0.003 M, red) and after addition of 1eq. DMAB (blue). 190 K, *l* = 0.4 mm, *T* = 190 K, toluene.

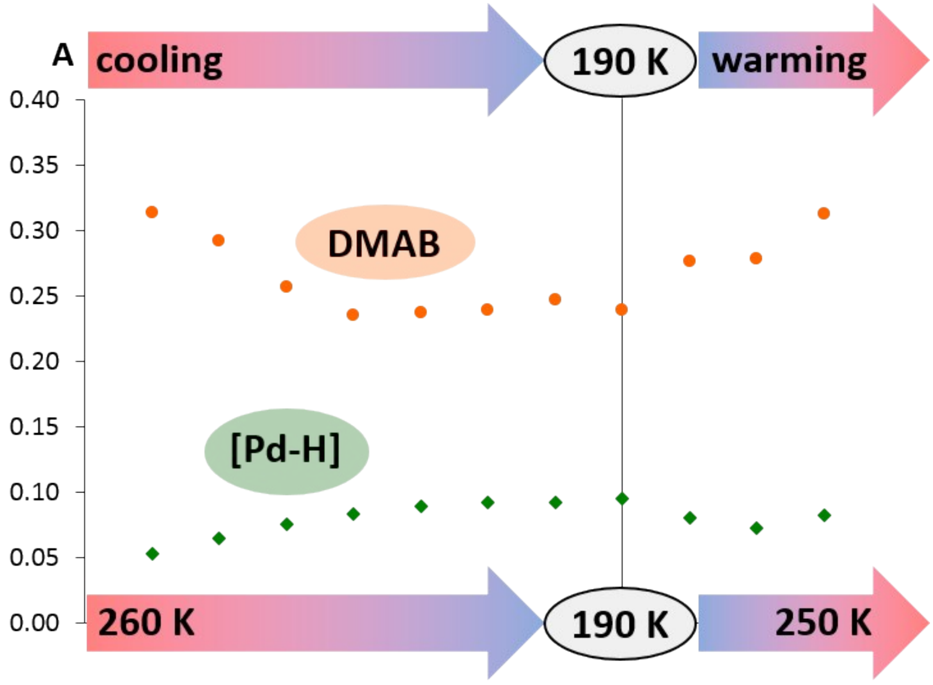

Figure S5. Diagram showing the temperature dependence of ν_{PdH} and ν_{BH} bands intensity in the mixture of ***4b*** (*c* = 0.01 M) and DMAB (*c* = 0.033 M) in toluene.

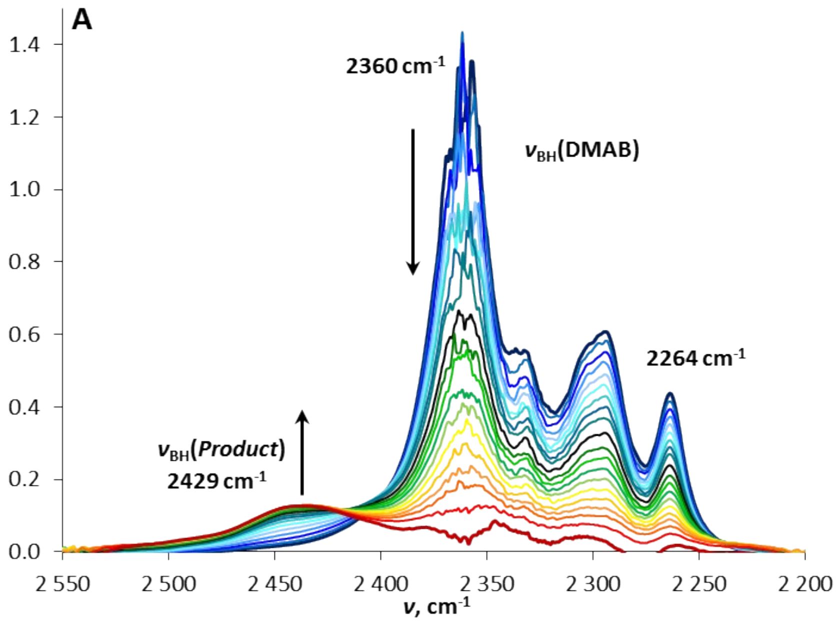

Figure S6. Time evolution of IR spectra of ***4a*** and DMAB mixture (1:5) in THF at 298K.

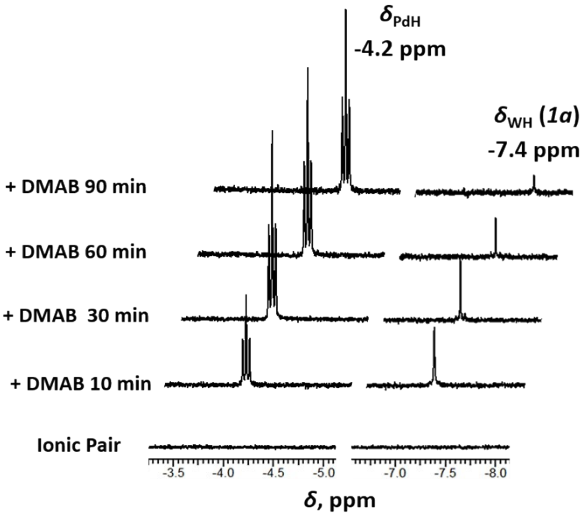

Figure S7. Time evolution ^{1}H NMR spectra (400 MHz, hydride region) of ***4a*** in the presence of excess DMAB (5 equiv). 290 K, THF-d_8.

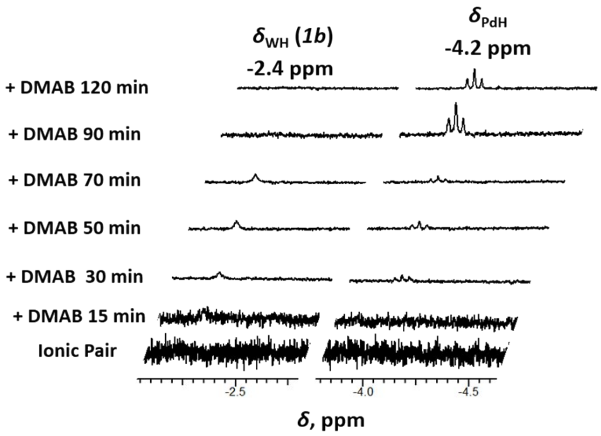

Figure S8. Time evolution of ^{1}H NMR spectra (400 MHz, hydride region) of ***4b*** in the presence of excess DMAB (5 equiv.). 290 K, THF-d_8.

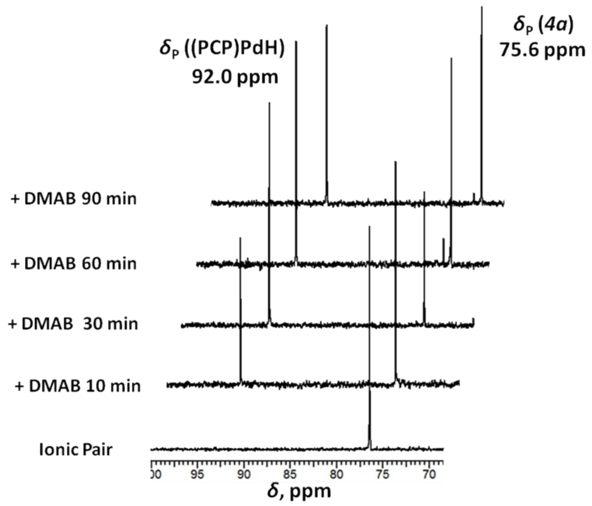

Figure S9. ^{31}P NMR spectra (161.98 MHz) of ***4a*** in the presence of excess DMAB (5 equiv.), monitoring in THF-d_8 at 290 K.

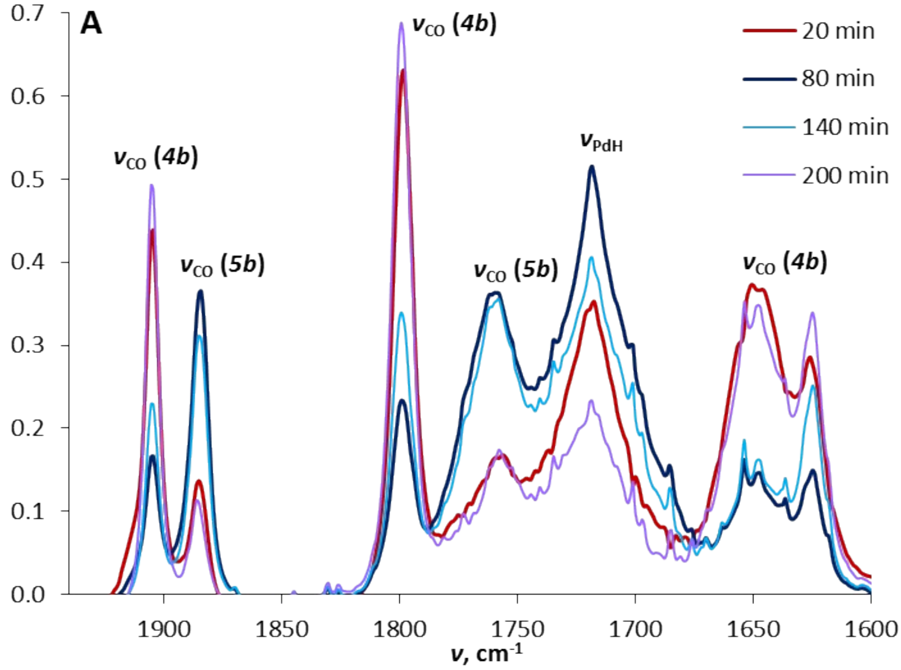

Figure S10. Time evolution of IR spectra of complex ***4b*** (c = 0.01 M) with DMAB (c = 0.033 M), 298 K, l = 2 mm, THF.

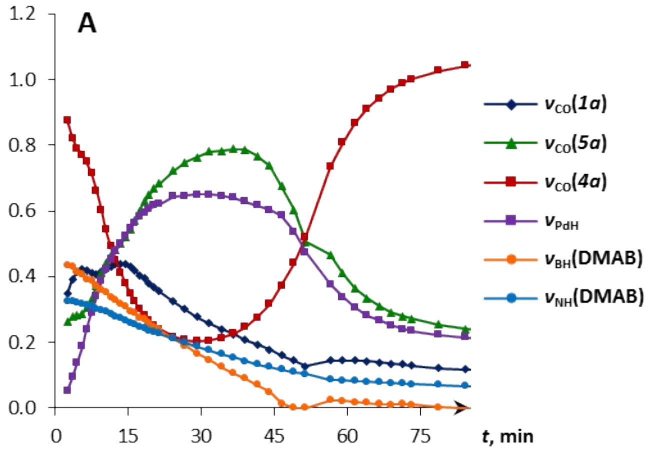

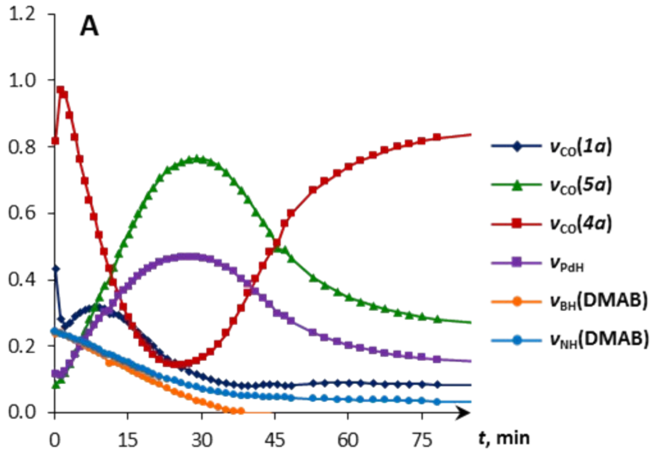

Figure S11. Kinetic curves obtained from IR spectra of dehydrogenation reaction of DMAB (5 equiv.) by ***4a*** at 298 K in THF. (a) Addition of DMAB to pregenerated ionic pair ***4a*** (*c* = 0.003 M); (b) simultaneous addition of DMAB, ***1a*** and (PCP)PdH (1:1, *c* = 0.005 M).

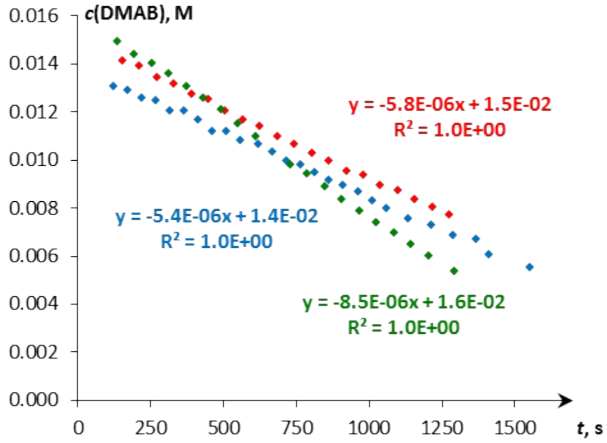

Figure S12. *c*(*t*) plots of DMAB dehydrogenation by ***4a*** (*c* = 0.003 M, red and blue) and upon the simultaneous mixing of three reagents (***1a*** + (PCP)PdH, 1:1, *c* = 0.005 M, green).

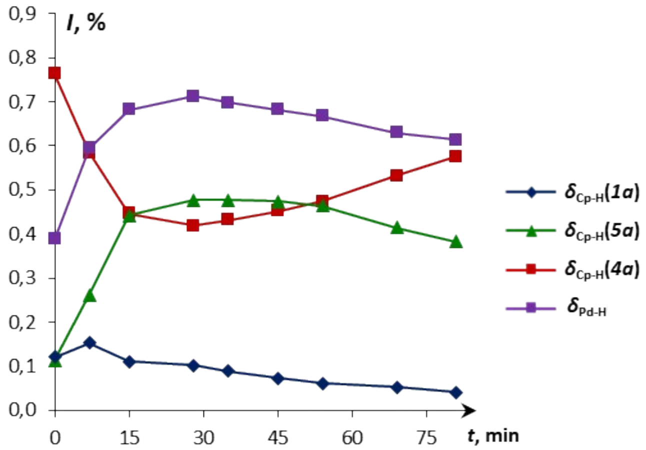

Figure S13. Normalized kinetic curves obtained from ^{1}H NMR spectra of dehydrogenation reaction of DMAB (5 eq.) by ***4a*** (*c* = 0.01 M), 293 K, THF-d_8.

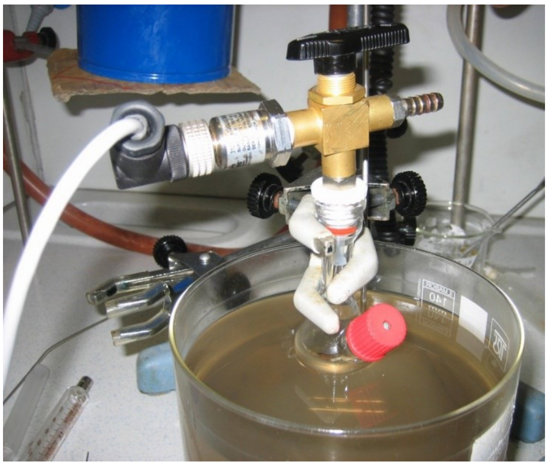

Man of the moon X103 is a device conceived by the University of Zaragoza (Spain) for monitoring the progress of reactions that evolve gases by measuring the pressure variation *vs.* time in closed reaction systems. More information about the features of the kit can be found at the following link: http://www.manonthemoontech.com/x103-gas-evolution.html.

Figure S14. “Man of the moon” device during a catalytic run.

Table S3. The catalytic performance of ***4a*** in the dehydrogenation of DMAB.

c_0(DMAB), M	Catalyst loading	*T*, K	TOF*, h^{-1}	Full conversion time, h	Initial rate, $M^{-1}\cdot s^{-1}$
0.015	***4a***, 20 mol%	296	**1.6**	5	$2.5\cdot10^{-6}$
0.015	***4a***, 20 mol%	313	**2.7**	4	$2.5\cdot10^{-6}$
0.03	***4a***, 10 mol%	296	**2.2**	4	$3.5\cdot10^{-6}$
0.06	***4a***, 5 mol%	313	**5.6**	6	$6.3\cdot10^{-6}$
0.15	***4a***, 2 mol%	313	**26.0**	3	$2.1\cdot10^{-5}$
0.1	***4a***, 10 mol%	296	**6.7**	2	$4.0\cdot10^{-5}$

*at half-conversion time

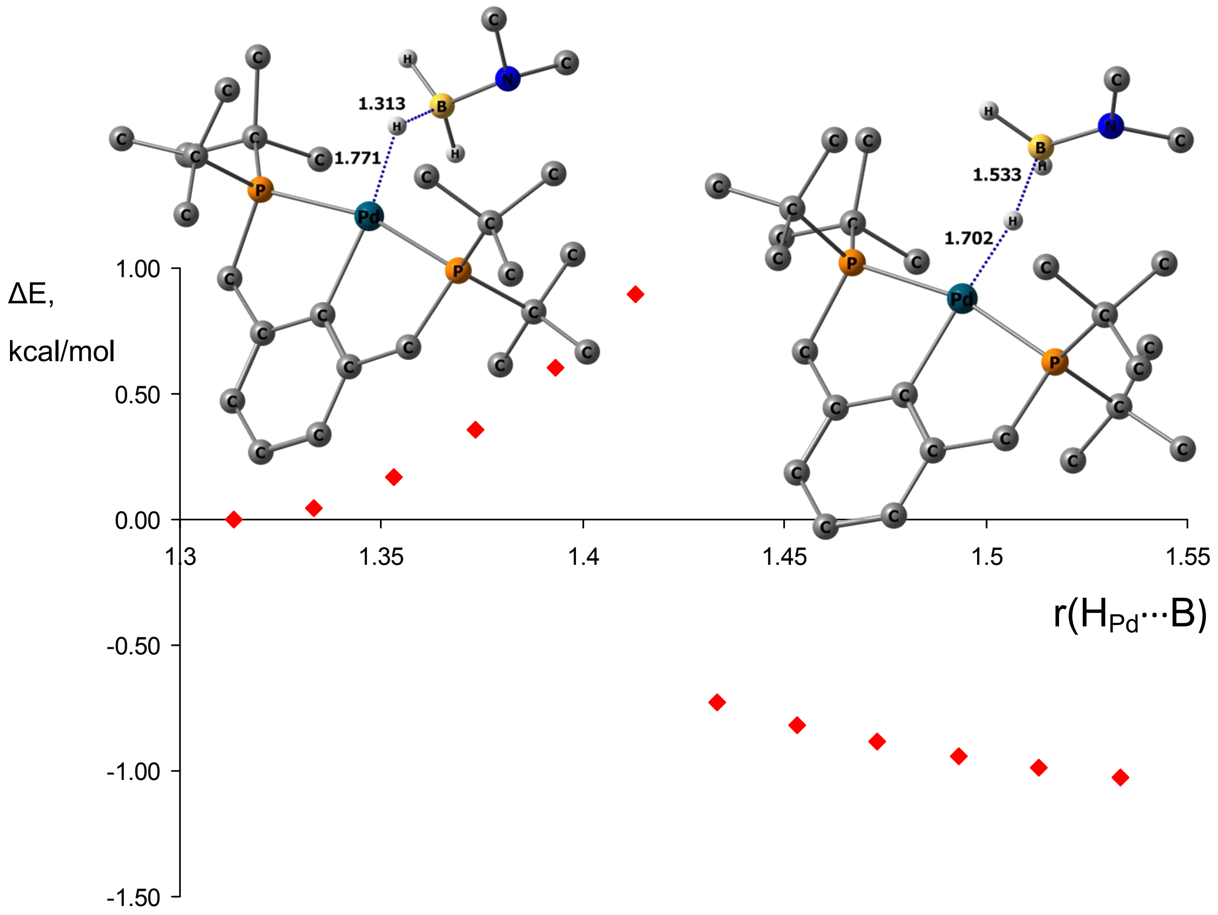

Figure S15. Relaxed PES scan for hydride transfer in complex ***6b***. TpWH(CO)$_3$ and hydrogen atoms, except BH/PdH are omitted.

TBAB dehydrogenation

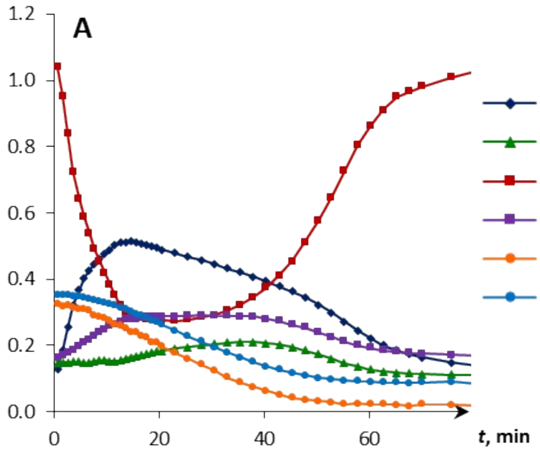

Figure S16. Kinetic curves obtained from IR spectra of dehydrogenation reaction of TBAB (5 eq.) by ***4a*** (*c* = 0.003 M), 298 K, THF.

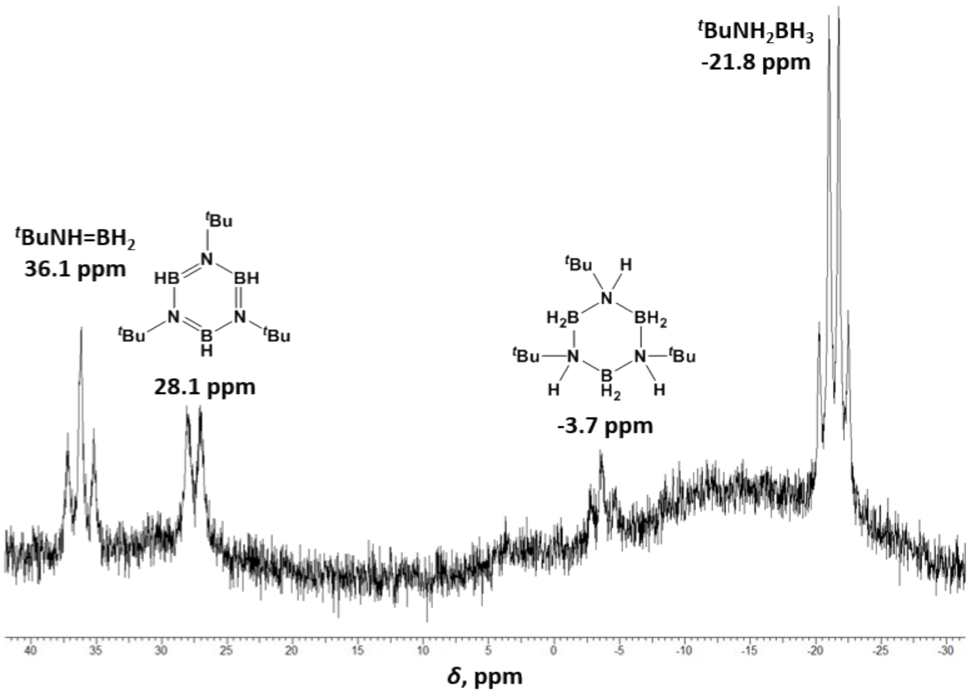

Figure S17. ^{11}B NMR spectrum (128.3 MHz) of TBAB (5 equiv.) in presence of ***4a*** an hour after mixing.

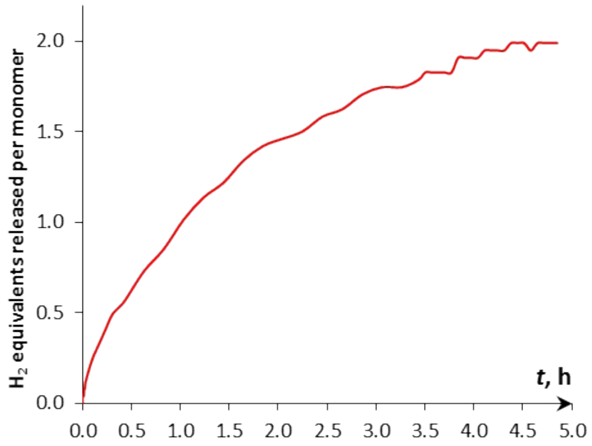

Figure S18. Kinetic curve of TBAB dehydrogenation by [CpW(CO)$_2$(μ-CO)···Pd(PCP)] (***4a***, 20 mol.%, c = 0.003 M) in THF at 296 K.

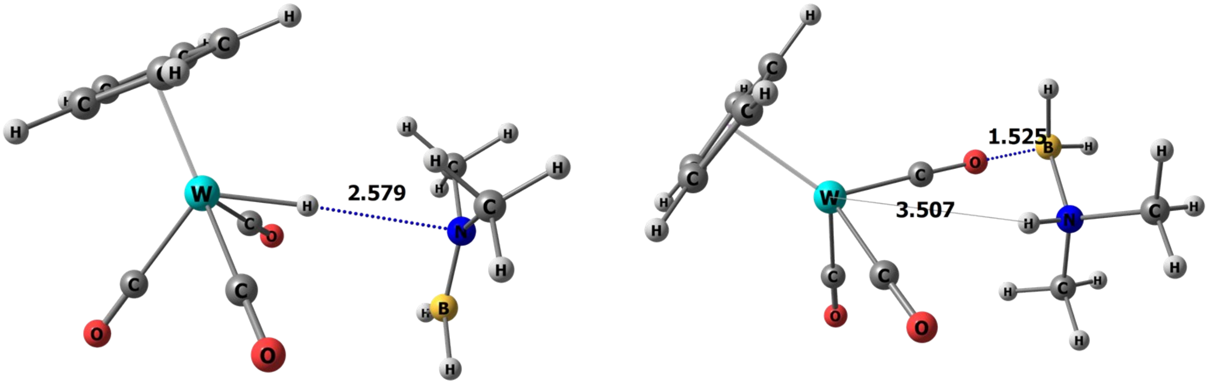

CpW(CO)$_3$H // H$_2$BNMe$_2$

E=0.0 E=+1.3

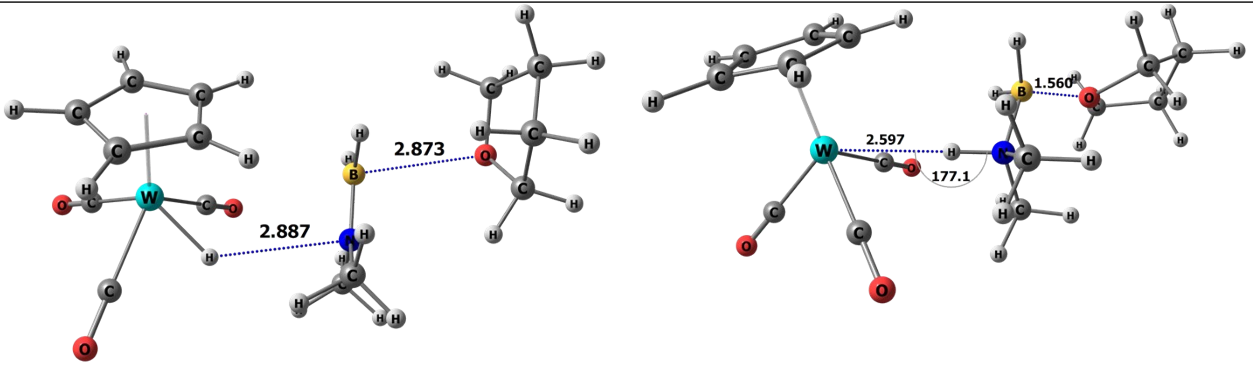

CpW(CO)$_3$H // H$_2$BNMe$_2$ // THF

E=0.0 E=-0.2

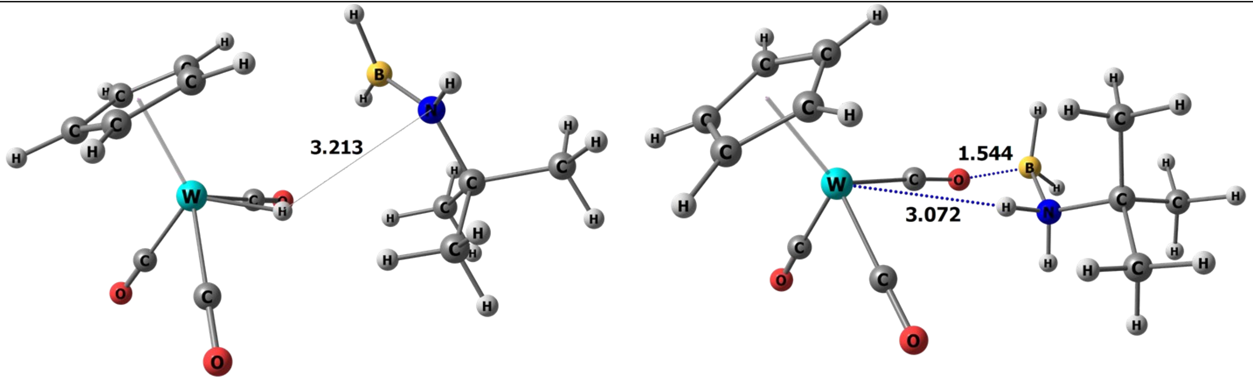

CpW(CO)$_3$H // H$_2$BNH^{t}Bu

E=0.0 E=+3.8

Figure S19. Optimized structures of neutral (right) and ionic (left) complexes of CpW(CO)$_3$H with H$_2$BNR$_2$ and stabilized by THF. The energies are in kcal/mol respective to the neutral pair.

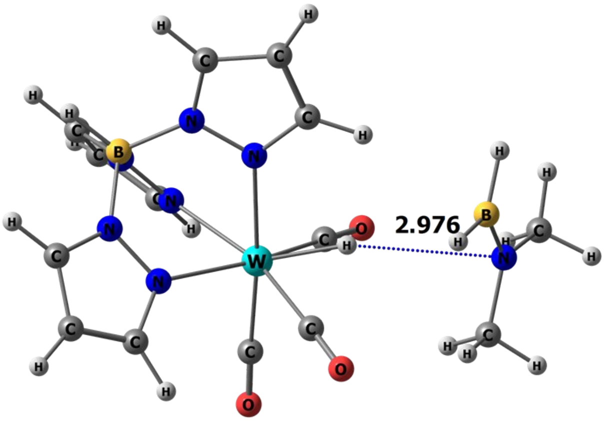

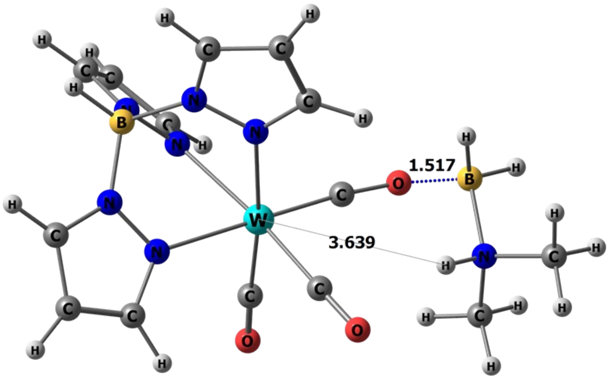

$TpW(CO)_3H$ // H_2BNMe_2

E=0.0

E=-1.7 kcal/mol

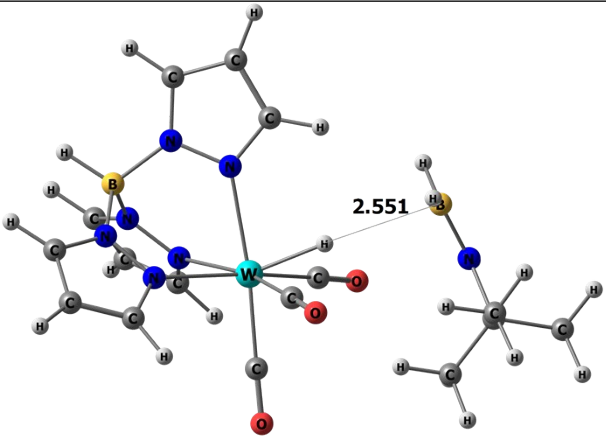

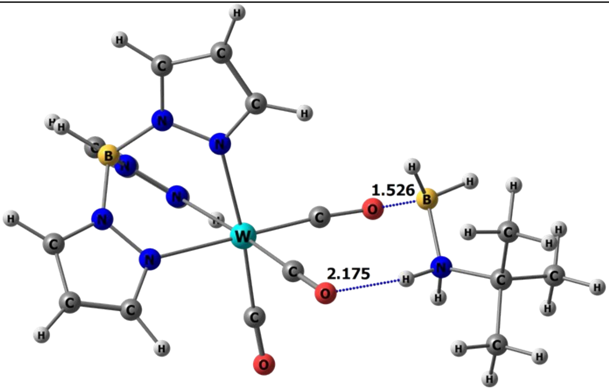

$TpW(CO)_3H$ // H_2BNH^tBu

E=0.0

E=+0.6

Figure S20. Optimized structures of neutral (right) and ionic (left) complexes of $TpW(CO)_3H$ with H_2BNR_2. The energies are in kcal/mol respective to the neutral pair.

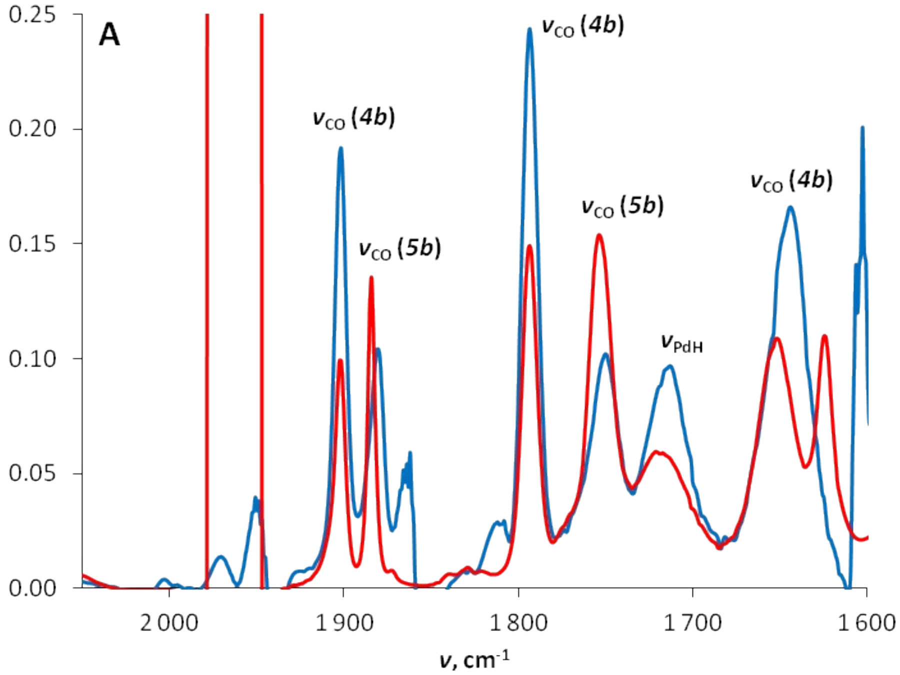

Figure S21. IR spectra of ***4b*** and DMAB mixture (1:1, *c*= 0.003 M) in THF and toluene, 190 K, *l* = 0.5 mm.

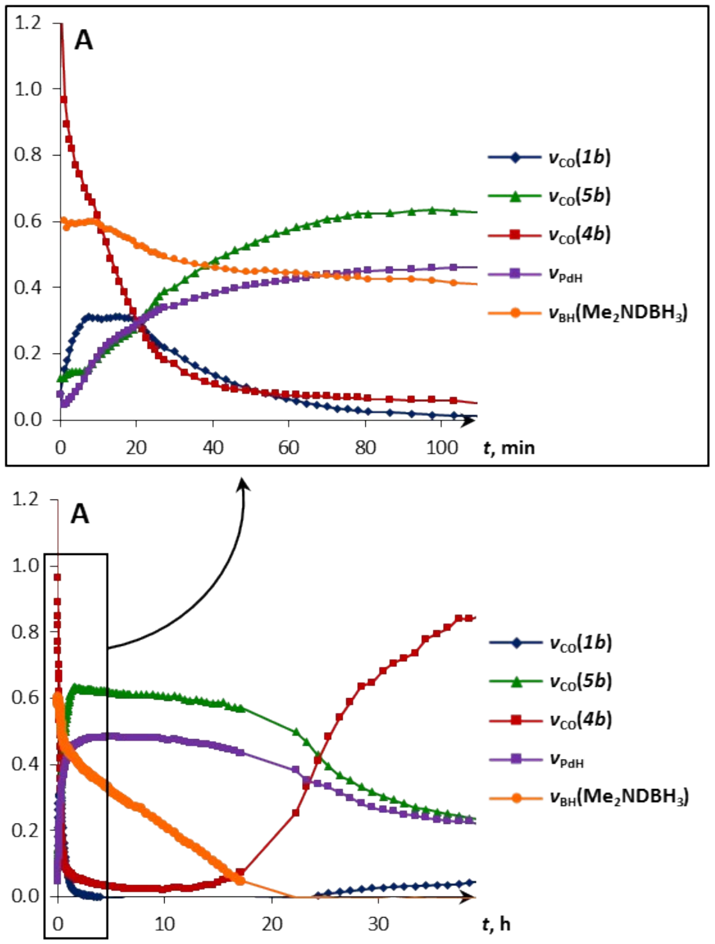

Figure S22. Kinetic curves obtained from IR spectra of dehydrogenation reaction of Me_2NDBH_3 (5 equiv.) by ***4a*** (*c* = 0.003 M), 298 K, THF. Initial (a) and full regions (b).

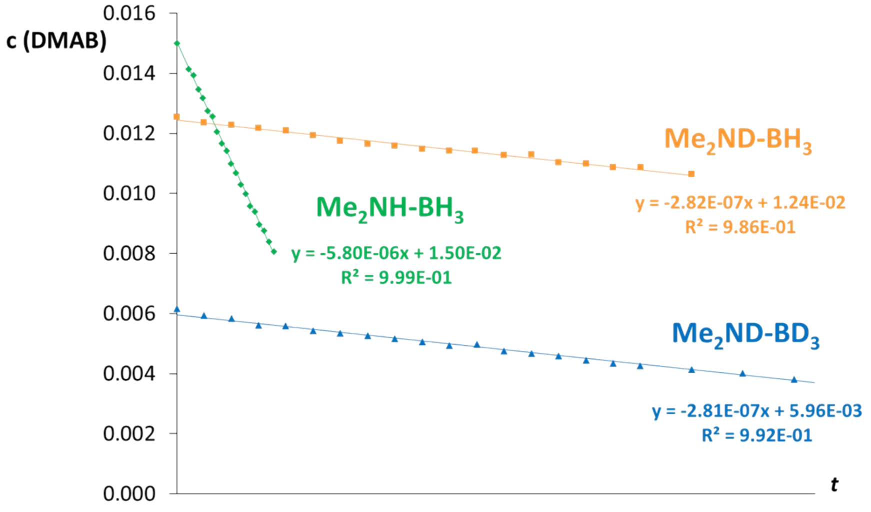

Figure S23. $c(t)$ plots obtained from IR spectra of 5 equiv. Me_2NDBH_3, Me_2NDBH_3 and Me_2NHBH_3 dehydrogenation by ***4a*** (c = 0.003 M), 298K, THF.

Kinetics analysis

Excluding the catalyst and substrate decomposition, the proposed reaction mechanism could be simplified to the following scheme:

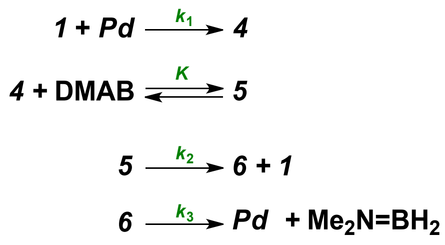

Pd = (PCP)PdH

The reaction between two neutral hydrides ***1*** and (PCP)PdH could be described as an irreversible process with pre-equilibrium[1]. The observed rate constant for this process is k_1. Since the rate of hydrogen evolution in the reaction between two neutral hydrides ($3{\cdot}10^{-4}$ M·c^{-1}; ***1***:***Pd*** = 1:1, c = 0.003 M, L = Cp) is higher than in DMAB dehydrogenation ($2.5{\cdot}10^{-6}$ M·c^{-1}; ***1***:***Pd***:DMAB = 1:1:5, c = 0.003 M, L = Cp), it could be considered that $k_1 > k_2$. The formation of ***5*** is an equilibrium process with an equilibrium constant $K = \frac{[\mathbf{5}]}{[\mathbf{4}][\mathrm{DMAB}]}$. The DFT-calculated activation energy values for stepwise proton and hydride transfer ($\Delta G^{\ddagger}_{PT}$ = 21 kcal/mol and $\Delta G^{\ddagger}_{HT}$ < 2 kcal/mol) indicate that $k_3 \gg k_2$, and the hydride transfer goes much faster than proton transfer. The shift of the equilibrium due to fast hydride transfer and evolution of hydrogen makes the subsequent reactions ***5***→***6***+***1*** and ***6***→***Pd***+**$Me_2N{=}BH_2$** almost irreversible. Thus, the following kinetic equations can be derived:

$r_1 = k_1{\cdot}[\mathbf{1}]{\cdot}[\mathbf{Pd}]$
$[\mathbf{5}] = K{\cdot}[\mathbf{4}]{\cdot}[\mathrm{DMAB}]$
$r_2 = k_2\,[\mathbf{5}]$
$r_3 = k_3\,[\mathbf{6}]$

Considering that proton transfer is rate-limiting stage, the overall reaction rate (r) equals to r_2:
$r = k_2 \cdot [\mathbf{5}] = k_2 \cdot K \cdot [\mathbf{4}] \cdot [\mathrm{DMAB}]$

For the initial stage, the experimentally observed DMAB consumption in presence of substrate excess is pseudo-zero order in DMAB and first order in catalyst ***4***. This could be approximated by the following equation:
$\boldsymbol{r = k_2 \cdot K \cdot c_0(\mathrm{DMAB}) \cdot [4] = k_{eff} \cdot [4]}$.

Table S4. Experimental rate constants for Me_2NHBH_3 (DMAB) and $^{t}BuNH_2BH_3$ (TBAB) dehydrogenation and corresponding activation free energies $\Delta G^{\neq}$ (kcal/mol).

Catalyst	Substrate	k_1, $M^{-1} \cdot s^{-1}$	k_2K, $M^{-1} \cdot s^{-1}$	$\Delta G^{\neq}$(**4**→**6**)	k_2, s^{-1}	$\Delta G^{\neq}$(**5**→**6**)
4a	DMAB	2.4 [1]	$5.7 \cdot 10^{-6}$	24.6	$1.7 \cdot 10^{-1}$	18.5
4a	TBAB	2.4	$5.7 \cdot 10^{-6}$	24.6	$1.0 \cdot 10^{-1}$	18.8
4b	DMAB	4.5	$1.5 \cdot 10^{-6}$	25.4	$3.1 \cdot 10^{-2}$	19.5

References:

[1] Osipova, E. S.; Belkova, N. V.; Epstein, L. M.; Filippov, O. A.; Kirkina, V. A.; Titova, E. M.; Rossin, A.; Peruzzini, M.; Shubina, E. S., *Eur. J. Inorg. Chem.* 2016, 1415-1424.
